# Horizontal transfer of plasmid-like extrachromosomal circular DNAs across graft junctions in Solanaceae

**DOI:** 10.1186/s43897-024-00124-0

**Published:** 2024-11-20

**Authors:** Aijun Zhang, Tingjin Wang, Lu Yuan, Yuxin Shen, Ke Liu, Bin Liu, Kexin Xu, Mohamed A. Elsadek, Yiting Wang, Liang Wu, Zhenyu Qi, Jingquan Yu, Mingfang Zhang, Liping Chen

**Affiliations:** 1 Department of Horticulture, College of Agriculture and Biotechnology, Zhejiang University, Hangzhou, 310058 China; 2 Department of Agronomy, College of Agriculture and Biotechnology, Zhejiang University, Hangzhou, 310058 China; 3 Institute of Virology and Biotechnology, Zhejiang Academy of Agricultural Sciences, Hangzhou, 310021 China

**Keywords:** Tomato, Goji, Distant grafting, EccDNA transfer, Pleiotropic traits, Asexual offspring inheritability

## Abstract

The transfer of genetic material between stocks and scions of grafted plants has been extensively studied; however, the nature and frequency of the transferred material remain elusive. Here, we report a grafting system involving woody goji as the stock and herbaceous tomato as the scion, which was developed using in vitro and in vivo approaches; the results confirmed horizontal transfer of multiple nuclear DNA fragments from donor goji cells to recipient tomato cells. Tomato tissues containing goji donor DNA fragments at or near the grafting junctions had a perennial-biased anatomical structure, from which roots or shoots were regenerated. Most of the fragments were plasmid-like extrachromosomal circular DNAs (eccDNAs) present in the regenerants derived from the cells and in their asexual offspring. Plants with transferred eccDNAs in regenerated roots or shoots (designated “Go-tomato”) were grown perennially and showed excellent agronomic performance. The present study provides new insights into the replication, expression, and potential function of eccDNAs in the pleiotropic traits of Go-tomato. Mobile eccDNAs offer evidence of stock-to-scion horizontal DNA transfer beyond chromosomes and organelles, thereby contributing to the molecular understanding of graft-induced genetic variation, evolution, and breeding.

**Supplementary Information:**

The online version contains supplementary material available at 10.1186/s43897-024-00124-0.

## Core

Genomic and RNA sequencing revealed that tomato scion cells and their regenerants harbor eccDNAs that were derived from goji stock through horizontal transfer and were heritable in asexual offspring. The present research provides direct evidence for the stock-to-scion transfer of nuclear DNA fragments in the form of plasmid-like circular DNAs across a graft junction.

## Gene and accession numbers

Raw data that support the findings of this study have been deposited in Genome Sequence Archive (GSA) at http://gsa.big.ac.cn under the accession number CRA013197. All the other data generated in this study are included in the article and additional files.

## Introduction

Grafting is an ancient agricultural technique that enhances the yield, quality, and resistance of grafted plants (Mudge et al. 2009; Wang et al. 2016, 2024; Zohary and Spiegel-Roy 1975). This technique typically involves joining a stem from one plant (the scion) to the root system of another plant (the stock). However, the potential of the asexual grafting process to transfer genetic material and the underlying mechanisms remain controversial and require further clarification.

Asexual transmission of cytoplasmic male sterility to grafted scions and their progeny was first observed in *Petunia* by Frankel as early as the 1950s (Frankel 1956, 1962). Subsequent studies have attempted to demonstrate transmission through grafting in diverse taxa such as eggplant (*Solanum melongena*, Solanaceae) (Hirata 1980), soybean (*Glycine max*, Fabaceae) (Hirata and Yagishita 1986), and pepper (*Capsicum* sp., Solanaceae) (Taller et al. 1998). In recent studies, organelles and nuclear chromosomes were shown to be horizontally transferred across the graft junctions in tobacco (*Nicotiana tabacum*, Solanaceae) (Fuentes et al. 2014; Stegemann and Bock 2009; Stegemann et al. 2012). However, other studies have yielded inconsistent and even controversial results, failing to comprehensively explain the various phenomena of graft-derived genetic variation.

In eukaryotic cells, some cellular DNAs exist independently of conventional chromosomes and organelles in the form of extrachromosomal circular DNAs (eccDNAs). These eccDNAs are heterogeneous in size, ranging from hundreds to several hundred thousand base pairs, and they can originate from various regions of the genome, particularly regions with highly repetitive sequences (Ling et al. 2021; Peng et al. 2022; Yang et al. 2022). Advances in sequencing techniques and bioinformatics have greatly enhanced our understanding of the breadth and diversity of eccDNAs. Unlike chromosomal amplicons, eccDNAs follow a non-chromosomal mechanism of inheritance; these molecules lack centromeres and segregate unequally into daughter cells, thereby enabling them to adapt rapidly to environmental changes (Peng et al. 2022). EccDNAs have high mobility and self-replication ability, and they show transcriptional activity, particularly under stress conditions (Bakhoum et al. 2018; Møller et al. 2018; Paulsen et al. 2018; Wang et al. 2021b; Wang et al. 2021; Zuo et al. 2022). Given the mobility of eccDNAs and their intercellular connections that facilitate the transfer of large molecules, we hypothesize that eccDNAs might play a role in cell-to-cell communication during the grafting process, potentially leading to phenotypic variation mediated by grafting. However, our understanding of the presence and abundance of eccDNAs in the stock and scion, their potential for mobility and transfer from the stock to scion, and their role within the recipient cells during grafting remains limited.

In the present study, we established a grafting system between the solanaceous species woody goji *Lycium ruthenicum* as the stock and herbaceous tomato *Solanum lycopersicum* as the scion, which produced regenerants in the scion stem tissue. By using sequencing approaches, we aimed to characterize horizontal transfer of genetic material, primarily eccDNAs, from donor goji cells to recipient tomato cells across the graft junctions and in subsequent regenerants. This transmission led to substantial pleiotropic traits in the tomato plant, which was designated as “Go-tomato.” Our findings provide new genomic insights into eccDNA exchange between heterologous and homologous cells in asexual grafts and their offspring in the form of plasmid-like circular DNAs, thus shedding light on the long-standing curiosity about graft-derived genetic variation.

## Results

### Establishment of a compatible intergeneric grafting system between goji and tomato

**Fig. 1:**
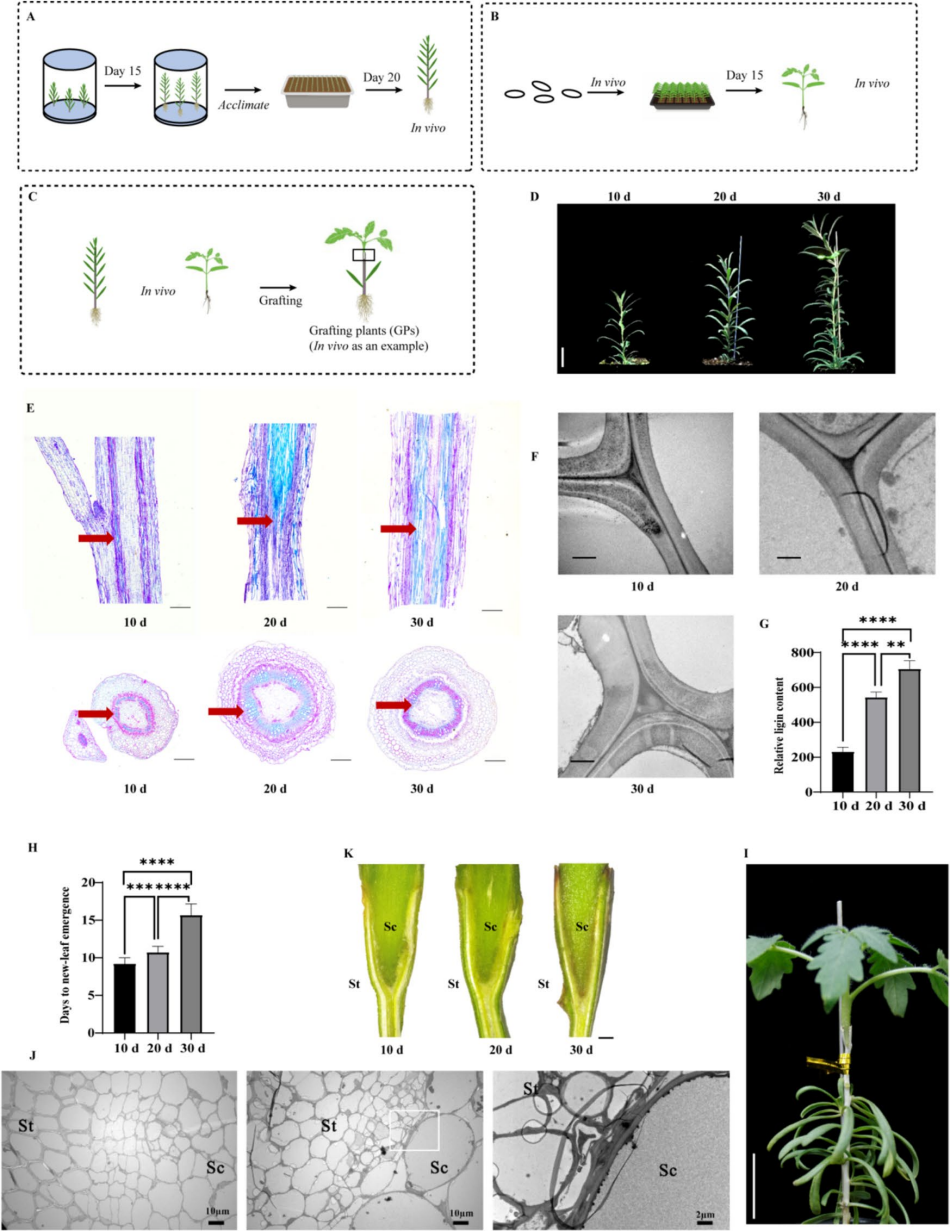
Successful establishment of a grafting system between distant Solanaceae genera. **A**–**C** Summary of the in vivo grafting experimental workflow used in this study. **A** In vitro grown goji seedlings were transplanted to an in vivo culture and acclimated for 20 d to serve as stocks for in vivo grafting. **B** In vivo tomato scions were obtained for subsequent grafting by transferring the germinated seeds to a hole tray filled with a moist matrix. **C** The in vivo grafting process. **D** Phenotype of goji transferred into the matrix for the indicated number of days. Scale bar = 2 cm. **E** Paraffin sections (top: longitudinal; bottom: horizontal) of goji stems at the indicated ages. Red arrows denote the xylem. Scale bars = 500 μm. **F** Transmission electron microscopy (TEM) images of the sub-microstructure of the goji cell wall at the indicated ages. Scale bars = 1 μm. **G** Relative lignin content of stocks at different stages. Differences between mean values were analyzed by Fisher’s exact test (**P < 0.01, ****P < 0.0001; *n* = 9). **H** Days for new leaves to emerge from the grafted rootstocks at different stages. Differences between mean values were tested by Fisher’s exact test (***P < 0.001, ****P < 0.0001; *n* = 9). I, Successful grafts with sprouting leaves. Scale bar = 2 cm. **J** TEM images illustrating the sub-microstructure of the grafting site of goji-grafted tomato after 15 d. Left, successful grafts; middle, unsuccessful grafts. Right: enlargement of the white box. Scale bars = 10 μm (left and middle) and 2 μm (right). St: goji rootstock, Sc: tomato scion. *n* = 9. **K** Longitudinal section of the graft junction of goji after grafting for 30 d using 10-, 20-, and 30-d goji stocks. St: goji rootstock, Sc: tomato scion. Scale bars = 1.52 mm

To definitively demonstrate the horizontal transfer of genetic materials and to overcome the analytical challenges associated with high homology among closely related species, we used goji stock (a wild woody plant) and a tomato scion (a cultivated herbaceous crop) within the Solanaceae family as models to establish a grafting system between distant species (Fig. 1A–C). Graft incompatibility due to the distant genetic relationship and anatomical differences between woody and herbaceous plants is the primary challenge in this approach. To mitigate this issue, we first investigated the effects of goji shoots at different developmental stages in vivo on graft incompatibility with tomato scions (Fig. 1D). Morphological and stem microstructure analyses of goji shoots revealed a continuous increase in xylem differentiation (Fig. 1E), cell wall thickening (Fig. 1F; Supplemental Fig. S1A), and a statistically significant increase in lignin content (Fig. 1G) during shoot development 10–30 d after in vivo transplantation.

To identify the optimal growth stage of goji stocks for successfully establishing a distant graft system, goji shoots at three developmental stages (10-, 20-, and 30-d transplantation) were tested as stocks. The grafting survival rate (evaluated based on scion sprouting) showed statistically nonsignificant differences between 10-d and 20-d stocks (Supplemental Fig. S1B). However, the grafting survival rate of 30-d stocks was notably reduced (Supplemental Fig. S1B). Scions grafted onto 10-d stocks sprouted more frequently than those grafted onto 20-d stocks, with approximately 45% more sprouting (Fig. 1H, I). In contrast, scions grafted onto 30-d stocks failed to sprout within 10 d of grafting (Fig. 1H). Sub-microstructure analysis revealed folded cell wall remnants in 30-d grafts, which is indicative of graft incompatibility, whereas successful grafts showed no distinct boundaries at the interface (Fig. 1J). Additionally, at 30 d after grafting, a brown necrotic layer was observed at the graft boundary, which was weakly developed in 20-d grafts and was almost absent in 10-d grafts (Fig. 1K). These results indicate that 10- and 20-d transplantation goji shoots, with a lower lignin content after in vivo transplantation, were more suitable for the adhesion of grafted cells in this experimental setup. Consequently, we selected 20-d goji stock for further grafting experiments because of its balanced lignin content and adequate toughness.

## Histological changes in tomato cells at the graft junctions and their subsequent organogenesis

**Fig. 2:**
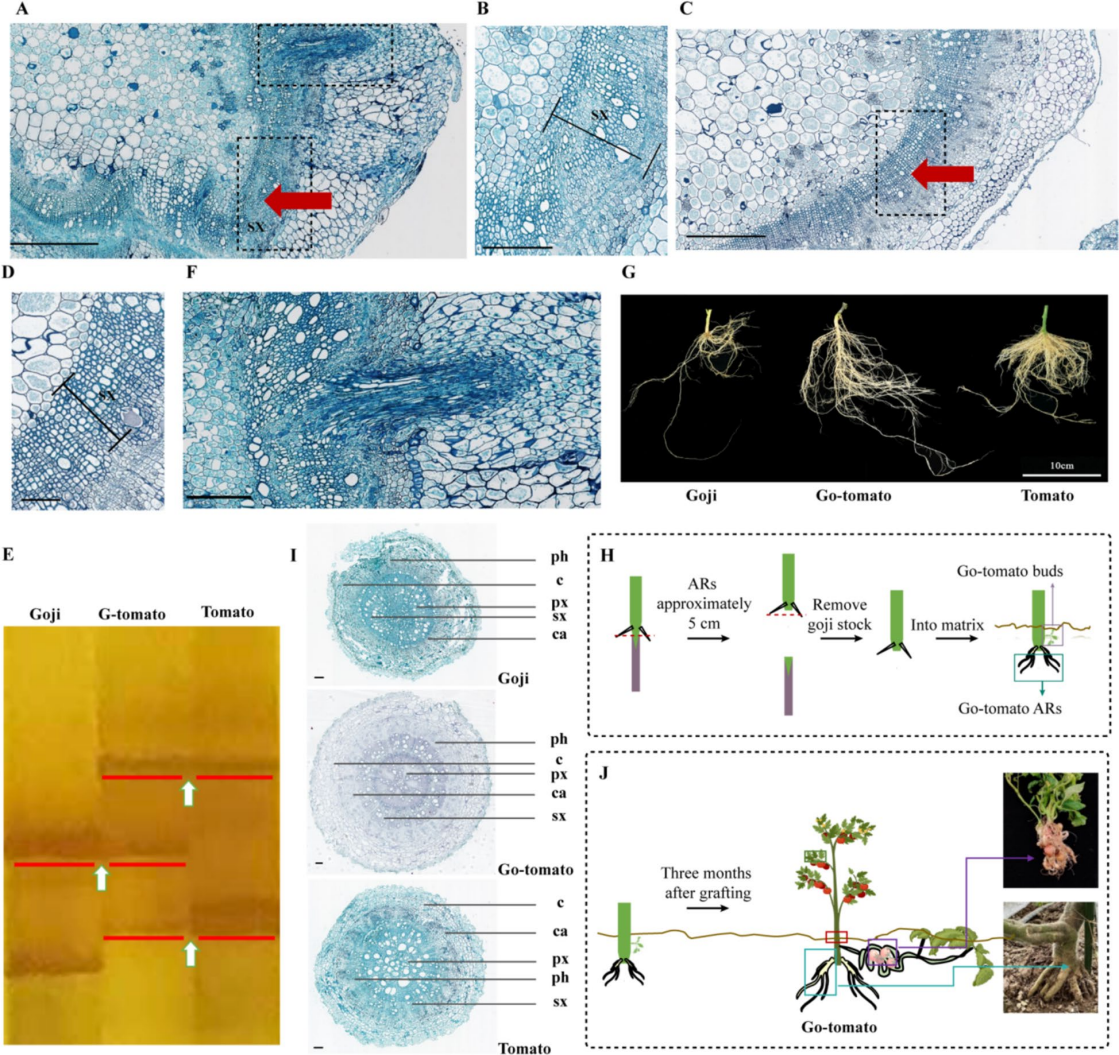
Morphological changes in tomato cells at the graft junctions and their subsequent organogenesis. **A–E** Optical microscopy images. **A** Transverse sections of the graft junctions at 30 d after grafting. The upper black box represents the origin of the adventitious roots, and the lower one represents morphological changes in tomato cells at the graft location. Arrows indicate the location for PCR sampling. Scale bar = 625 μm. **B** Enlarged image of the black boxes in A showing morphological changes in tomato cells at the graft location. Scale bar = 200 μm. **C** Transverse sections of the graft junctions of T-SPs at 30 d after grafting. The black box represents morphological changes in tomato cells at the graft location. Arrows indicate the location for PCR sampling. Scale bar = 625 μm. **D** Enlarged image of the black box in C representing the morphological changes in tomato cells at the graft site. Scale bar, 125 μm. **E** SRAP analysis of the tomato tissue at the healing site of grafting. Goji, goji control; Tomato, tomato control; G-tomato, tomato tissue at the healing site of grafting. Red bars and white arrows indicate co-dominant markers of goji, tomato, and G-tomato. **F** Enlarged image of the black box showing the origin of the adventitious roots in (**A**). Scale bar = 200 μm. **G** Morphology of the adventitious roots of goji, “Go-tomato,” and tomato plants (from left to right). Scale bar = 10 cm. **H** Schematic of adventitious organ formation in Go-tomato. The organs included adventitious roots and adventitious buds. **I** Transverse sections of the adventitious roots of goji, Go-tomato, and tomato. c, cortex; ca., cambium; px, primary xylem; sx, secondary xylem; ph, phloem. Scale bars = 125 μm. **J** Summary of Go-tomato formation after grafting. The blue box represents the regenerated adventitious root system from the tomato tissue across the graft junction of Go-tomato. Images are of representative seedlings at 3 months after grafting. Red box: Stem segment near the graft junction in Go-tomato. Purple box: Regenerated buds on Go-tomato plants. Green box: Go-tomato leaves

To establish the extent of healing activity at 30 d after in vivo grafting, toluidine blue staining was performed on paraffin sections obtained from the graft junctions of *g*rafted *p*lant*s* (GPs) and *t*omato *s*elf-grafted *p*lant*s* (T-SPs). We observed complete healing of the grafts (Fig. 2A and C), thus indicating that the sampling time exceeded the duration necessary to establish a vascular system network between the stock and scion (Martínez-Ballesta et al. 2010; Roberts et al. 2010). Strikingly, optical microscopy demonstrated that GPs displayed a continuous vascular cambium and a wider secondary xylem as compared to T-SPs (Fig. 2A–D), which suggested that grafting enhances the differentiation capacity of the cambium and secondary xylem development.

To investigate the factors contributing to the enhanced differentiation capacity of the cambium, we assessed whether genetic information was transferred from the stock to the scion during grafting adhesion. Sequence-related amplified polymorphism (SRAP) markers were used to confirm polymorphisms in goji cells, tomato cells, and the grafted tomato cells across the graft junctions (Fig. 2A, red arrow). A shared band with goji was detected; however, no specific band was detected in tomato plants (Fig. 2E). This finding suggests that certain DNA factors from goji might be transferred into the scion tomato cells, resulting in histological changes at the grafting union.

Adventitious roots were observed to originate from the cell layer in the scion tomato tissue at 30 d after grafting (Fig. 2F), which showed phenotypic variations (e.g., color and thickness) compared to non-grafted tomato adventitious roots (Fig. 2G). After the length of adventitious roots reached approximately 5 cm, the goji stock was removed from the graft junction, and the plants with adventitious roots were transplanted into the matrix (Fig. 2H). The regenerated adventitious roots were more robust than those of goji and scion tomato (Fig. 2G) and histologically developed in a tomato-biased manner (Fig. 2I). The height of T-SPs was greater than that of GPs and those with adventitious roots, with a statistically nonsignificant difference in the early stages after grafting. At around 70 d after grafting, plants with adventitious roots were statistically significantly taller than GPs but still shorter than T-SPs. From day 84 after grafting, the height of plants with adventitious roots was comparable to that of T-SPs, with both plant types becoming taller than GPs (Supplemental Figs. S1C and D). The chlorophyll content of the aboveground parts was statistically significantly higher in plants with adventitious roots than in T-SPs, thus indicating increased photosynthetic capacity of the former plants (Supplemental Fig. S1D).

Regenerated buds emerged above the adventitious roots in the matrix, with a frequency exceeding 10% after at least 3 months of growth; these buds exhibited stolon traits similar to those of goji (Fig. 2H and J, Supplemental Fig. S1E). Interestingly, these regenerated buds grew underground and bore several tomato-like fruits, with some small branches occasionally growing upward (Fig. 2J). However, after examining 14 buds from different plants, no mature seeds were found in the underground fruits (Supplemental Fig. S1E). Remarkably, plants with unique phenotypes in regenerated adventitious roots or buds had an extended growth cycle; they flourished for approximately 24 months in the field as compared to the typical 8-month growth cycle of T-SPs. These plants were successfully cultivated perennially with excellent performance (particularly high yield and fruit quality) in the temperate regions of Zhejiang and Shandong and the plateau areas of Qinghai Province, China, which are characterized by low temperatures and a wide diurnal temperature range (Supplemental Figs. S1F and G). This dramatic extension in the duration of tomato growth was attributed to the perennial trait acquired from goji. These plants were designated “Go-tomato,” and the *a*dventitious *r*oots of “*G*o-tomato” (G-ARs) were selected as materials for subsequent phenotypic variation and molecular genetic analyses.

## Horizontal transfer of DNA fragments occurs across the graft junctions to tomato plants

**Fig. 3:**
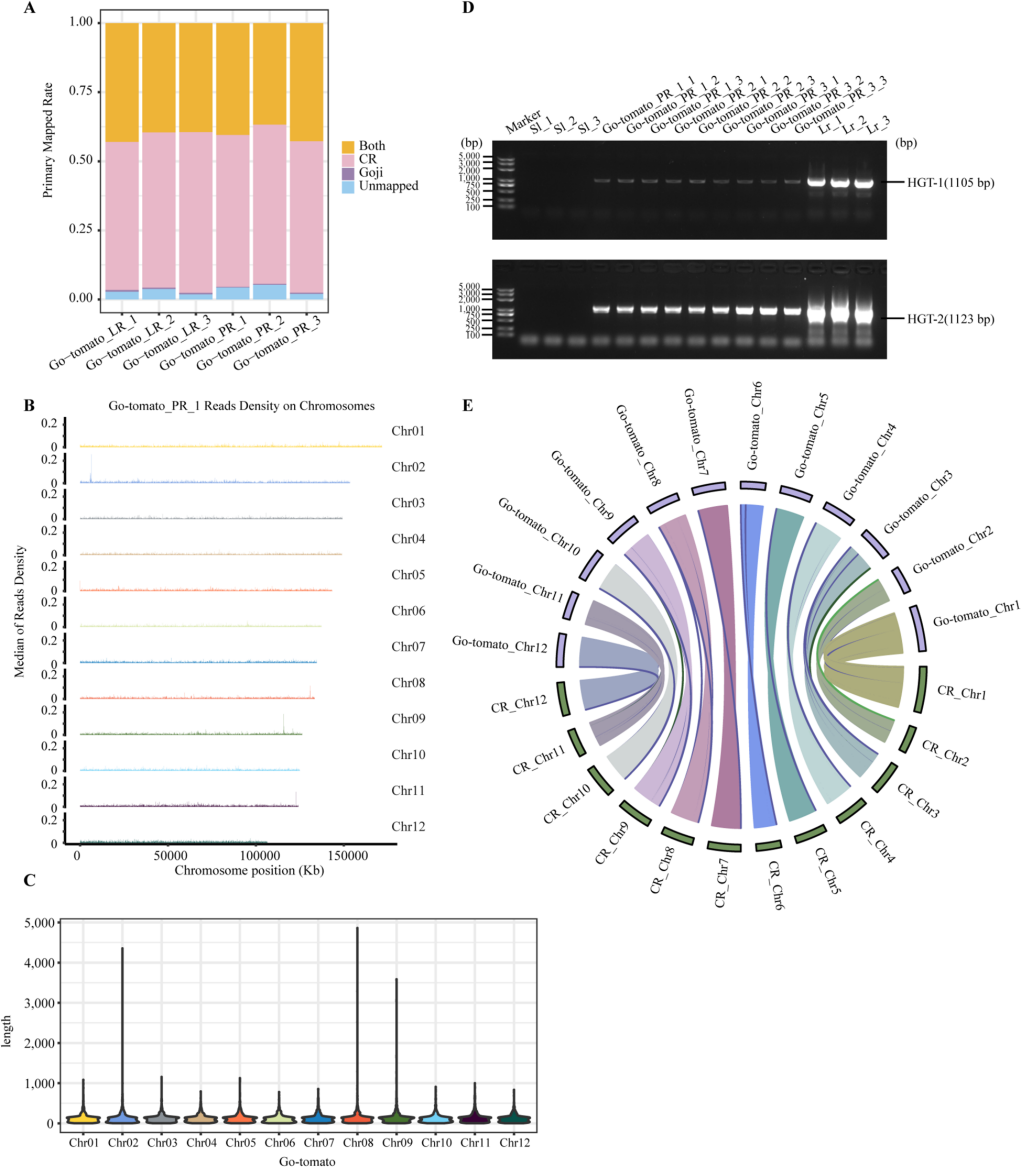
Transfer of goji DNA fragments to tomato occurs across a graft junction. **A** Primary mapping rates of resequencing reads to the reference genome for the primary and lateral roots obtained from Go-tomato_PR_1–3 and Go-tomato_LR_1–3 samples. PR: primary root; LR: lateral root. 1–3 represent three biological replicates. The reference genomes include the genomes of both goji and tomato. Pink (tomato) represents the proportion of the resequencing data aligned specifically with the tomato CR genome. Purple (goji) represents the proportion of the resequencing data aligned specifically with the goji genome. Pink + yellow (tomato + both) represent the resequencing data aligned with the tomato genome. Purple + yellow (goji + both) represent the resequencing data aligned with the goji genome. Blue (unmapped) represents the resequencing data that could not be aligned with either genome. **B** Median read density of Go-tomato_PR_1 mapped to the goji genome. The window length is 100 kb. Median read density represents count per window length; Chr indicates the chromosome where the resequencing data are mapped; and length represents the length of the sequences mapped to the chromosome. **C** Mapping results of the Go-tomato genome to the goji genome. Go-tomato represents the combined sequences from six samples (Go-tomato_PR_1–3 and Go-tomato_LR_1–3). **D** PCR amplification of HGT-1 (1105 bp) and HGT-2 (1123 bp) in tomato (*Sl*), goji (*Lr*), and Go-tomato_PR samples. HGT refers to genetic material transfer. 1–3 represent three biological replicates. Labels 1, 2, and 3 represent three technical repeats; the lengths of genetic material transfer fragments amplified using different pairs of primers are shown. **E** Distribution of features along the Go-tomato and CR genomes. Rings indicate 12 chromosomes of Go-tomato (Go-tomato_Chr1*–*Go-tomato_Chr12, upper semicircle) and CR (CR_Chr1*–*CR_Chr12, lower semicircle). The center shows the synteny relationships between the chromosomes of Go-tomato and CR, which are displayed as connecting lines

To investigate whether genetic material transfer occurs in G-AR, whole-genome resequencing analysis was performed on G-AR samples regenerated from the grafted tomato cells across the graft junctions (Supplemental Fig. S2A). Three biological replicates were prepared for each root tissue (primary root and lateral root of G-AR), resulting in six sequenced samples: Go-tomato_PR_1–3 and Go-tomato_LR_1–3 (Fig. 2J). These samples collectively yielded approximately 1,502,129,780 high-quality paired-end reads (Supplemental Table S1). After filtering reads aligned to the tomato CR (*Solanum lycopersicum* cv. Condine Red) genome, each read was uniquely aligned with the goji reference genome (Gautier et al. 2018). More than 300,998 fragments from each Go-tomato sample were extracted from the mapping interval of the goji nuclear genome (Fig. 3A–C; Supplemental Figs. S2B and 3A; Supplemental Tables S2–4). A total of 15,941 fragments were common to all six samples, with 25 fragments exceeding 1 kb (Supplemental Table S4). We further validated these findings through PCR analysis of all primary root samples (Supplemental Table S17). Interestingly, DNA fragments designated HGT-1 (1105 bp) and HGT-2 (1123 bp) were consistently identified across all Go-tomato samples and *Lr* (goji), albeit with varying signal intensities; however, *Sl* (tomato) did not contain detectable goji DNA (Fig. 3D). These results confirmed that goji DNA fragments were transferred to tomato. Go-tomato therefore contains genetic materials from goji, which were horizontally transferred by grafting.

To assess the frequency of DNA fragment transfer, PCR validation was extended to another set of regenerated adventitious roots from 24 grafted plants, 20 of which contained detectable goji DNA fragments (Supplemental Fig. S3B), which indicated a high frequency of genetic material transfer. Analysis of the regenerated primary and lateral roots showed that the transferred DNA fragments were maintained during mitosis (Supplemental Fig. S3C). PCR analysis of four parts of the same Go-tomato plant (Fig. 2A and J) revealed that goji DNA fragments were not detected in the upper leaves, but were found in the adventitious roots of goji, the stem segment near the graft junction, and the tomato tissues across the graft junction (Supplemental Fig. S3D). These findings confirmed that genetic material transfer occurred between heterologous cells across the graft junction and subsequently between homologous cells in grafted stems and their regenerated organs, which further indicated that the transferred fragments were mobile and could replicate.

To exclude the possibility that Go-tomato is a chimera plant and to determine the origin of these mobile DNA fragments after grafting, BLAST analysis was performed for goji-specific sequence reads in Go-tomato samples showing alignment to the nuclear genome. Only approximately 0.5% of these reads were mapped to organellar genomes, with only 0.15–0.22% of the sequences potentially matching the mitochondrial genome of *Lr* sequenced in this study (Supplemental Fig. S4A) and 0.01–0.15% of the sequences matching the chloroplast genome (Supplemental Table S5). Genomic collinearity analysis showed differences between the mitochondria of Go-tomato and *Lr*, while the mitochondrial genomes of Go-tomato and tomato were nearly identical, thus indicating no recombination of goji and tomato mitochondria (Supplemental Figs. S4B and C). Chloroplast DNAs from goji and tomato were distinguishable, and specific chloroplast fragments from goji were absent in the Go-tomato genome (Supplemental Fig. S4D). Collectively, these findings confirmed that goji nuclear DNA fragments were transferred to tomato, thereby excluding the possibility of cell-to-cell transmission of the entire organelle genome and the formation of a chimeric tissue through graft regeneration of goji and tomato cells (Supplemental Figs. S4C and D). The measurement of DNA content also indicated that Go-tomato and tomato have an equivalent genome size, further excluding the possibility of Go-tomato being a chimera (Supplemental Fig. S5).

To characterize the form of the goji DNA fragments after transfer across the junction, we performed long-read PacBio HiFi sequencing on tomato CR and G-AR samples (Supplemental Table S6). Two nuclear genomes were compared to identify potential inter-nuclear chromosomal arrangements using SyRI (Goel et al. 2019), and the results showed good collinearity with the tomato CR genome and the G-AR genome (Fig. 3E). These findings indicate that the transferred goji DNA fragments were not integrated into the tomato chromosomal genome, thus suggesting that their potential form as extrachromosomal DNA fragments.

## Distinct transmission of eccDNA and its multiple functions in Go-tomato

**Fig. 4:**
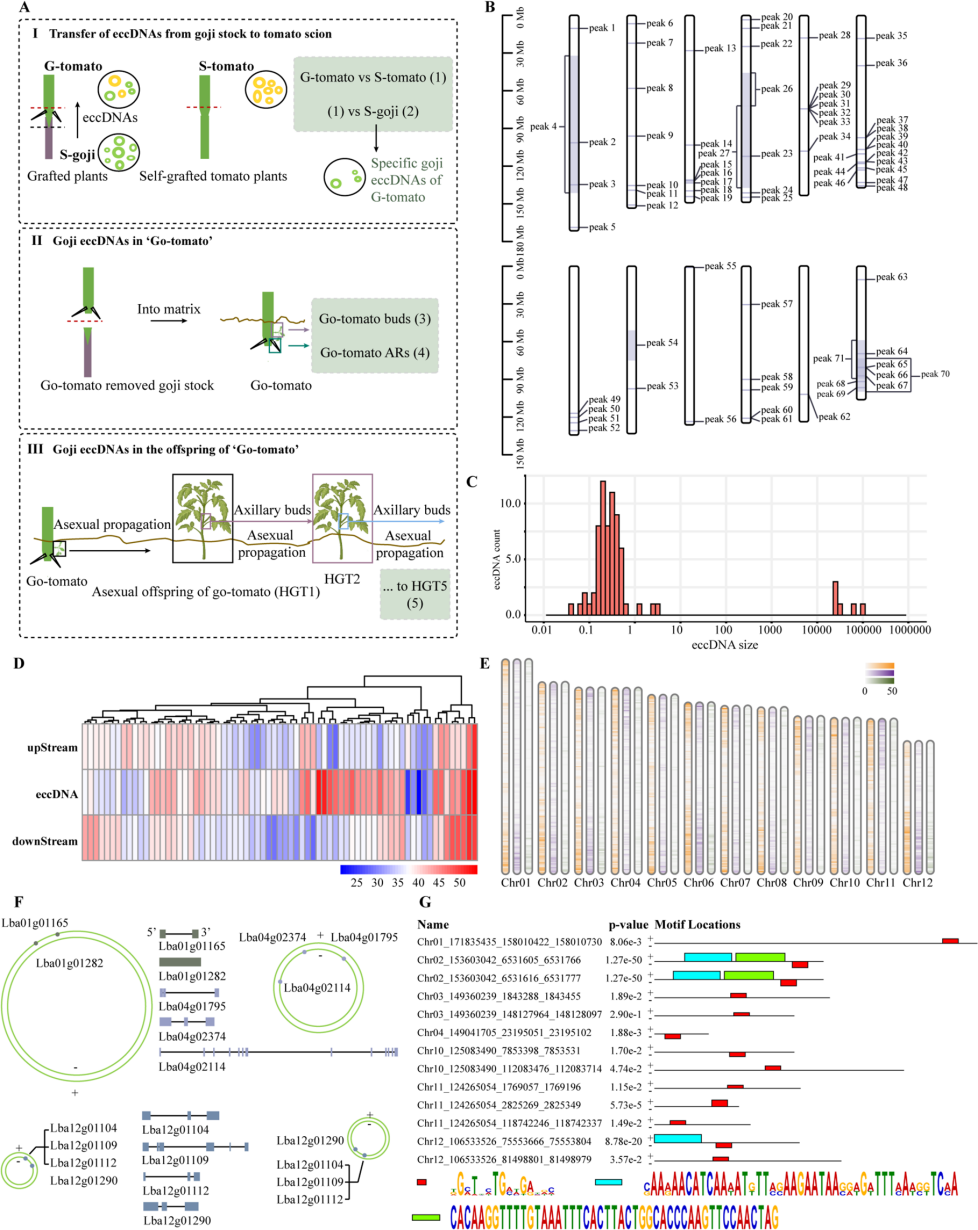
Mapping of eccDNAs across the goji genome and their regulatory roles in Go-tomato. **A** Schematic of eccDNA sampling and data comparison. I, Transfer of eccDNAs from the goji stock to the tomato scion. Three samples were taken for eccDNA sequencing, namely grafted tomato scions (G-tomato), grafted goji stocks (S-goji), and self-grafted tomato scions (S-tomato). The sampling sites of G-tomato and S-tomato are above the red line, while the sampling site of S-goji is below the black line. The large black circle represents eccDNAs. Yellow and green circles represent eccDNAs of S-tomato and S-goji, respectively; G-tomato includes eccDNAs from both S-tomato and S-goji. We compared eccDNAs in G-tomato with those in S-tomato (step 1) to obtain goji-specific eccDNAs transferred from the goji stock to the tomato scion (step 2). II, To define goji eccDNAs in Go-tomato, we performed eccDNA sequencing in samples from Go-tomato buds (step 3) and adventitious roots (step 4) to identify goji eccDNAs present in the grafted plants. III, To define goji eccDNAs in Go-tomato offspring. Go-tomato buds were asexually propagated to the fifth generation and assigned HGT5 (step 5). **B** Overall chromosomal distribution of 71 eccDNAs across the goji genome in G-AR-2. Peak IDs represent the identifier number of eccDNA in this analysis. Dark blue: Position of each eccDNA; light blue: length of each eccDNA. **C** Distribution of the eccDNA size in the G-AR-2 sample. **D** Heatmap showing GC content distribution near eccDNA sequences in G-AR-2. **E** Density and distribution of eccDNAs across the 12 goji chromosomes in G-tomato, S-goji, and S-tomato. Orange, purple, and green represent G-tomato, S-goji, and S-tomato, respectively. The window size for all three samples is 1 Mb. Heatmaps show the distribution of eccDNAs in S-goji, G-tomato, and S-tomato. **F** Structure of eccDNAs and the associated goji genes on eccDNAs in G-AR-2. The box represents coding sequences of different goji genes. **G** Conserved sequence elements identified using MEME suite in plasmid-like eccDNAs detected in the samples of asexually produced offspring. Red, blue, and green symbols represent motifs identified in 13 unique eccDNAs maintained in asexual offspring; the predicted motif consensus is shown in Supplemental Table S15

Considering that eccDNA exists outside the primary chromosomes and plays diverse roles in the genome, we used Circle-seq (Deng et al. 2022; Huang et al. 2022) on G-AR with two biological replicates to confirm the formation of goji DNA fragments after horizontal gene transfer (Fig. 4A). This method involves enzymatic degradation of linear DNA and enrichment of eccDNAs resistant to exonuclease activity. These samples yielded 379,960,746 high-quality paired-end reads. A total of 99 eccDNAs were identified in both samples, with 4 overlaps and 21 partial overlaps, thus indicating a high degree of uniqueness of eccDNAs to each sample, even within the same tissue type. This finding was consistent with the results of Zhuang et al. (2024) (Supplemental Figs. S6A and B; Supplemental Tables S7–10). Taking G-AR-2 as an example, we further examined the characteristics of eccDNAs, each of which was aligned with the goji reference genome (Fig. 4B; Supplemental Tables S7 and S8); this led to the identification of 71 unique eccDNAs, predominantly ranging from 0.1 to 1 kb with high GC content as compared to upstream and downstream sequences (Fig. 4C and D; Supplemental Tables S9 and S11). These findings were validated using PCR with both convergent and divergent primer pairs (Supplemental Fig. S6C). We compared the common transferred goji DNA fragments in the resequencing data and found that 2848 fragments overlapped with Go-tomato eccDNAs (Supplemental Table S12) and the 71 unique eccDNAs detected in G-AR-2 overlapped with 3780 genes (Supplemental Table S13).

Several genes associated with key biological pathways were identified. For instance, three genes related to the anthocyanin biosynthesis pathway were identified: *Lba01g01282* (*MATE*), *Lba12g01948* (*F3H*), and *Lba12g02047* (*DFR*). *Lba12g02047* (*DFR*) catalyzes the reduction of flavonol to produce colorless anthocyanin; thus, this gene is critical for anthocyanin accumulation. *Lba12g01948* (*F3H*) encodes a flavanone 3-hydroxylase, which is expressed together with chalcone synthase and chalcone isomerases in flavonoid biosynthesis (Tanaka and Ohmiya 2008). *Lba01g01282* (*MATE*) is annotated as a gene encoding a MATE efflux family protein with anthocyanin transport function (Gomez et al. 2009, 2011; M’Mbone et al. 2018; Marinova et al. 2007). Two genes related to cell division and differentiation were identified: *Lba01g01165* (*WOX5*) and *Lba04g02114* (*NOT9A*); these genes are essential for columella stem cell maintenance in the root meristem and regulation of cell differentiation, respectively (Burkart et al. 2022; Leonardo et al. 2021). Two WRKY28 transcription factor genes associated with senescence, namely *Lba04g02374* and *Lba12g01290*, were also identified (Hinckley and Brusslan 2020; Tian et al. 2020). *Lba12g01109* (*AN3*), which is crucial for plant growth and development, particularly in root meristem development (Xiong et al. 2020), was also identified (Fig. 4F; Supplemental Table S13). Thus, our findings indicate that DNA fragments transferred from goji by grafting exist as eccDNAs in tomato cells, with multiple gene functions in Go-tomato.

The biogenesis of transposon-derived eccDNAs is associated with plant stress response, thus indicating that active transposable elements may provide substrates for DNA integration, displacement, recombination, and gene capture, thereby enabling plants to rapidly develop novel stress responses. For instance, cold stress represses *Nightshade* eccDNA formation in potato (Esposito et al. 2019), while drought stress increases *Rider* eccDNA formation in tomato (Benoit et al. 2019). These observations show that stress influences the dynamics of eccDNA formation, thereby potentially affecting plant adaptation to environmental challenges. To further comprehend the impact of grafting on the dynamics of eccDNA formation, we sequenced eccDNAs in samples from S-goji and goji controls. Although the goji stock could produce eccDNAs, the length and count of eccDNAs were reduced in response to grafting (Supplemental Figs. S6D and E). These results highlight the potential of stress cues (such as grafting) to downregulate eccDNA formation.

To specifically differentiate goji eccDNAs transferred from the goji stock to the tomato scion, we conducted Circle-seq analyses on three distinct tissues: self-grafted goji stock (S-goji), self-grafted tomato scion (S-tomato), and grafted tomato scion (G-tomato) (Supplemental Table S9). In addition to the overlap of eccDNAs with S-tomato, 8284 eccDNAs were identified in G-tomato, and 39 of these eccDNAs were aligned with S-goji eccDNAs (Fig. 4A and E; Supplemental Table S14). Moreover, 19 of these eccDNAs were aligned with the eccDNA data from the fifth generation of asexual buds (Fig. 4A; Supplemental Table S14). To determine the presence of conserved sequences in eccDNAs maintained in asexual offspring, we analyzed the sequence features of 19 eccDNAs by using MEME suite. Because of file size limitation for data upload, we excluded six excessively long sequences. We found potentially conserved regions in the remaining 13 unique eccDNAs maintained during asexual propagation (Fig. 4G); the predicted motif consensus is shown in Supplemental Table S15. These conserved regions might have potential roles in the replication origin of eccDNAs. Overall, these findings not only confirm that goji eccDNAs from the goji stock were indeed horizontally transferred to the tomato scion after grafting, but also imply that eccDNAs can be partially maintained in asexual offspring.

## Goji eccDNA transfer induces pleiotropic traits

**Fig. 5:**
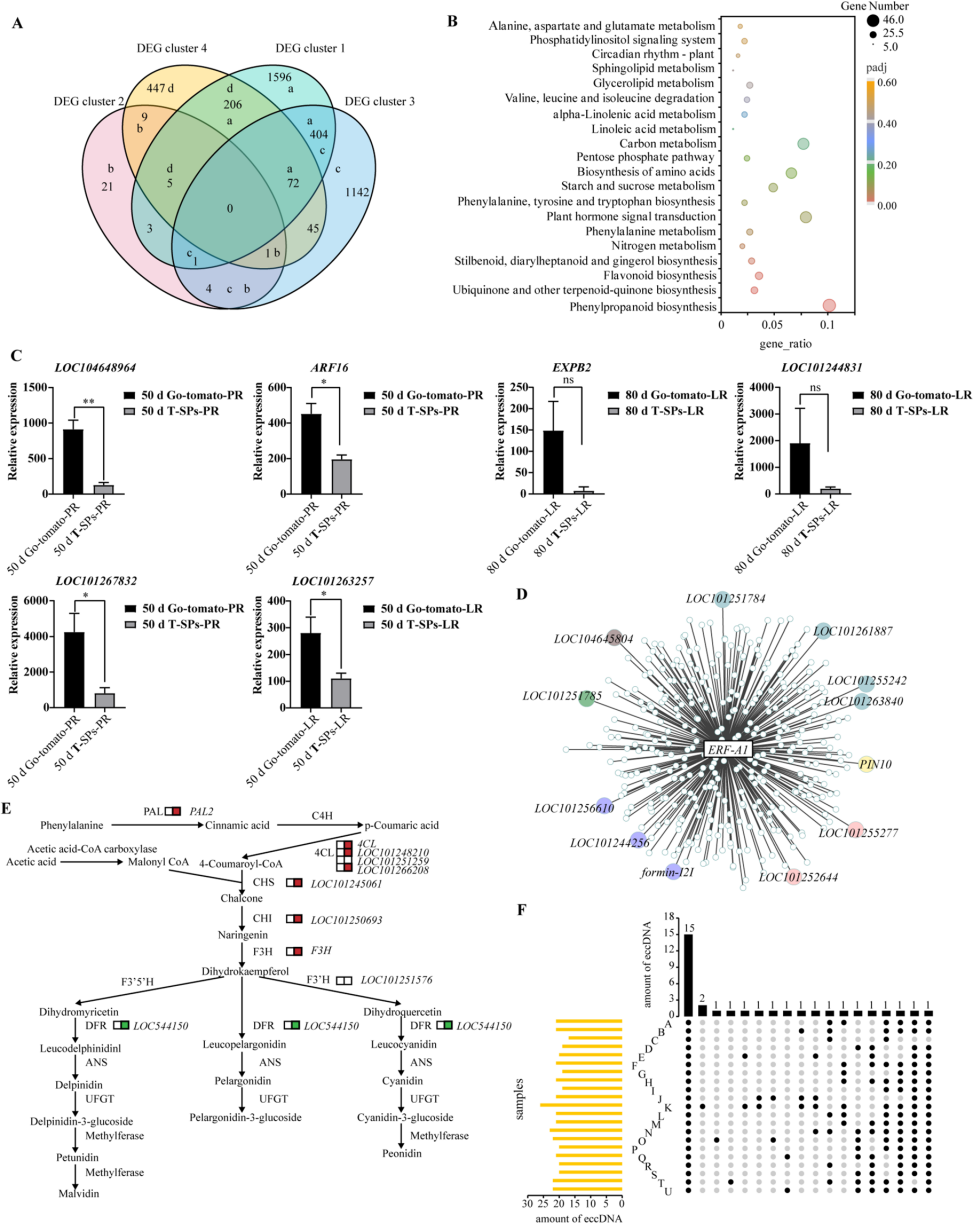
Grafting-induced changes in gene expression in the tomato regenerated G-AR system. **A** Gene Ontology enrichment analysis of three different conditions. DEG cluster 1 indicates DEGs between the regenerated primary roots of Go-tomato and T-SPs at 50 d after grafting; Cluster 2 indicates DEGs between the regenerated primary roots of Go-tomato and T-SPs at 80 d after grafting; Cluster 3 indicates DEGs between the regenerated lateral roots of Go-tomato and T-SPs at 50 d after grafting; Cluster 4 indicates DEGs between the lateral roots of Go-tomato and T-SPs at 80 d after grafting. **B** Kyoto Encyclopedia of Genes and Genomes (KEGG) pathway enrichment scatterplot. The plot indicates KEGG pathway enrichment analysis of a in panel (**A**). **C** Expression analysis of genes related to cell division and differentiation, and nutrient absorption and transport. (*P < 0.1, **P < 0.01; *n* = 9). **D** Weighted gene co-expression network analysis for the regulatory network. **E** Analysis of DEGs associated with phenylpropanoid and flavonoid biosynthesis pathways. Red and green indicate upregulated and downregulated genes, respectively. **F** Transcriptome data and eccDNA sequence mapping results. **A**–**J** represent samples CR-50 d-LR-1-3, CR-80 d-LR-1-3, CR-50 d-PR-1-3, and CR-80 d-PR; **K**–**U** represent Go-tomato-50 d-LR-1-3, Go-tomato-80 d-LR-1-3, Go-tomato-50 d-PR-1-2, and Go-tomato-80 d-PR-1-3

To analyze the influence of eccDNA transfer on gene expression, we conducted RNA-seq analysis on 24 samples and elucidated gene expression patterns in the primary and lateral roots of T-SPs and G-AR sampled at two developmental stages (50 and 80 d after grafting), with three biological replicates for each tissue type. All reads were uniquely aligned with those of tomato. At 50 d, a total of 2287 unigenes, including 1443 upregulated and 844 downregulated unigenes, exhibited differential expression in the primary roots of the G-AR system as compared to that in T-SPs (Fig. 5A, DEG cluster 1). Similarly, in lateral roots, 1669 unigenes showed statistically significant differential expression, with 1047 upregulated and 622 downregulated unigenes (Fig. 5A, DEG cluster 3). At 80 days after grafting, the primary roots of G-AR had 89 differentially expressed unigenes, with 60 upregulated and 29 downregulated unigenes (Fig. 5A, DEG cluster 2) as compared to those of T-SPs. In lateral roots, 785 differentially expressed unigenes were identified, including 326 upregulated and 459 downregulated unigenes (Fig. 5A, DEG cluster 4) (Supplemental Fig. S7A).

To understand the molecular mechanisms underlying the enhanced development of the G-AR, we focused on DEG clusters 1 and 3 at the 50 d time point. Two AP2/ERF transcription factors, namely *LOC104648964* and *LOC101267832*, were identified in DEG cluster 1 (Fig. 5A). These genes are associated with cell division, xylem and phloem tissue genesis, and cambium activity, which collectively regulate lateral growth in stems and roots (Hoang et al. 2020). The auxin response factor *ARF16*, which is responsible for adventitious root initiation and root cap cell differentiation (Gawroński et al. 2021; Wang et al. 2005), exhibited statistically significant differences in the expression levels in primary roots between Go-tomato and T-SPs (Fig. 5C). This result suggests a pivotal role of *LOC104648964*, *LOC101267832*, and *ARF16* in cell division and differentiation in G-AR primary roots after grafting. The co-expression network analysis of the differentially expressed genes revealed that the genes regulating cambium development, anthocyanin biosynthesis, lignin biosynthesis, and senescence were co-expressed with *LOC101267832* (Fig. 5D). Furthermore, we identified genes associated with the absorption and transport of essential nutrients. Two phosphate transporters, namely *LOC101256919* (*PHO1*) and *LOC101263257* (*PHT4;4*) with functions in root-shoot and ascorbate transport, were statistically significantly downregulated and upregulated by 2.45- and 2.53-fold, respectively, in G-AR lateral roots at 50 d after grafting (Fig. 5A, DEG cluster 3). These changes contribute to the establishment of systemic phosphate homeostasis, facilitate plant reproductive development, and increase the levels of reduced ascorbate in leaves (Luan et al. 2018; Miyaji et al. 2015). Collectively, these results highlight the enhanced capacity of G-AR lateral roots to absorb and transport, exceeding that of T-SPs (Zohary and Spiegel-Roy 1975).

A subset of 477 genes were common between DEG clusters 1 and 3 (Fig. 5A). Two predicted regulatory sites were identified within the promoter region of certain genes, most notably for *LOC104648964* (*ERF-1*), which is closely associated with cell division and differentiation and was upregulated. *LOC101245039* (*4CL1*), involved in phenylpropanoid and flavonoid biosynthesis pathways, was also upregulated in the primary and lateral roots (Fig. 5E). These findings suggest that *ERF-1* and *4CL1* are probably the target genes affected by horizontal gene transfer.

To further elucidate the temporal effects of G-AR at distinct developmental stages, we analyzed G-AR transcriptomes at 80 d, with a focus on DEG clusters 2 and 4 (Fig. 5A). A set of 15 genes were common to the two clusters, whereas no common genes were identified among the 477 genes encompassing DEG clusters 1 and 3 (Fig. 5A and Supplemental Fig. S7B). In DEG cluster 2 (Fig. 5A), 44 genes were identified, including nine genes that overlapped with the DEGs in cluster 1 (Fig. 5A). These genes were primarily enriched in pathways related to the biosynthesis and metabolism of specialized metabolites, such as monoterpenoid, phenylpropanoid, and phenylalanine. In contrast, genes exclusive to DEG cluster 1 (Fig. 5A) were enriched in plant hormone signal transduction and amino acid biosynthesis and degradation pathways. DEG cluster 4 comprised 785 genes, including 118 genes shared with DEG cluster 3 (Fig. 5A). Genes in DEG cluster 3 were enriched in plant–pathogen interaction and amino acid biosynthesis pathways, while genes in DEG cluster 4 were associated with various metabolic pathways (Fig. 5B and Supplemental Fig. S7C). Following a thorough analysis of DEG clusters 2 and 4, we found two genes with established roles in plant growth and development. The expansin gene *EXPB2* and the cell elongation factor *LOC101244831* are involved in cell wall-bound peroxidase activity, oxidative stress tolerance, and secondary cell wall formation (Han et al. 2015; Hossain et al. 2012). In DEG cluster 4, both *EXPB2* and *LOC101244831* were strongly upregulated. This upregulation trend was consistently maintained in DEG cluster 2 (Fig. 5A and C). These findings highlight the potential significance of genes related to plant growth and development in promoting the enhanced growth observed in the G-AR system post-grafting.

*AUXIN RESPONSE FACTOR 6 (ARF6)* and *ARF8*, known to interact physically with DELLAs, play specific roles in repressing phloem proliferation and inducing cambium senescence during the xylem expansion phase (Ben-Targem et al. 2021; Gawroński et al. 2021). *LOC101259615* (*ARF6a*) expression increased in primary roots (Fig. 5A, DEG cluster 2). In contrast, *LOC100301945* (*ARF8b*) expression was downregulated at 50 d as compared to that in the primary roots of T-SPs, as observed in DEG cluster 1 (Fig. 5A). These findings collectively suggest that G-AR primary roots initiated the process of cambial senescence by 80 d after grafting.

To comprehensively evaluate the overall transcriptional impact of eccDNAs, we conducted a comparative analysis of the transcriptome data with eccDNA sequences. eccDNAs present in G-AR samples had unique transcripts—a phenomenon conspicuously absent in T-SP roots (Fig. 5F and Supplemental Table S16). Among these unique transcripts, *mRNA1*, derived from a segment of *Lba12g01384*, is believed to be involved in regulating stress responses. *mRNA2* was attributed to a portion of *Lba05g01225*, which encodes ribosomal protein S4 localized within the chloroplast (Supplemental Table S16). Our data indicate that eccDNAs are transcriptionally active and express functional mRNAs that play a role in regulating gene expression.

Taken together, these results indicate that variations in genes related to cell division and differentiation, nutrient absorption and transport, and plant growth and development contribute to distinct phenotypic alterations observed in Go-tomato roots. Transcriptomic changes observed in the G-AR were consistent with histological alterations and pleiotropic traits evident in both G-AR and Go-tomato. Consequently, the thriving phenotype of G-AR is closely related to the upregulation of genes involved in cell division and differentiation (Fig. 2B and C), while the vigorous growth of Go-tomato is associated with genes responsible for nutrient absorption/transport and plant growth and development (Fig. 2D).

## Discussion

The success of plant grafting depends on the reconstruction of plasmodesmata between heterologous cells, which facilitates the exchange of genetic information (Gautier et al. 2018; Habibi et al. 2022). Studies on genetic information exchange between the stock and scion in the grafting process have primarily focused on proteins (such as transcription factors) and RNAs (mRNAs and small RNAs) that are crucial for regulating diverse developmental and physiological processes (Chen et al. 2006; Liu et al. 2023; Zhu et al. 2007). However, during cell division and plant regeneration, particularly through asexual reproduction, these genetic materials appear to be diluted over time and do not fully explain the mechanisms underlying the inheritance and maintenance of graft-induced genetic variation. Hence, it is necessary to investigate graft-derived genetic variation at the DNA level. Recent evidence suggests that genetic material is transferred between species through close contact; this phenomenon is known as horizontal gene transfer (or lateral gene transfer), and it is recognized as the driving force for evolution and adaptation (Bock 2010; Shi 2016; Wickell and Li 2020). Plant grafting can be successfully performed across different varieties, species, and genera, which provides a structural basis for horizontal gene transfer (Davis and Wurdack 2004; Mower et al. 2004; Thyssen et al. 2012). Studies on the transfer of mobile genetic material between the stock and scion through the grafting junction have predominantly utilized tobacco as a model, which limits broader applicability because of the close genetic relationship between the stock and scion (Fuentes et al. 2014; Gurdon et al. 2016; Stegemann and Bock 2009; Stegemann et al. 2012).

In the present study, we established a grafting system by using two different Solanaceae genera: the herbaceous tomato with an undeveloped cambium as the scion and the perennial woody goji with a developed cambium and secondary growth as the stock. This approach excluded the possibility of cell-to-cell transmission of the entire chloroplast or mitochondrial genomes, as confirmed by mitochondrial genome sequencing and chloroplast-specific sequence screening. While a previous study monitored nuclear fusion through grafting that led to sexual reproduction (Fuentes et al. 2014), our present experimental design precluded possibilities of cell fusion and cell chimerism. We confirmed the mobility of large-scale nuclear DNA fragments across the graft junctions; moreover, the transferred nuclear DNA fragments exhibited potential preference, mobility, and replicability, as validated by PCR analyses with different Go-tomato samples or different tissues from the same Go-tomato sample.

To comprehensively analyze the nature of goji DNA fragments transferred into the tomato genome, we conducted long-read PacBio HiFi sequencing on G-AR; the results showed good collinearity with the tomato CR genome. We found that these mobile DNA fragments were not integrated into the recipient tomato genome; instead, they existed as extrachromosomal DNA fragments within the recipient tomato cells. EccDNAs are circular DNAs derived from, but independent of, chromosomes, and show a wide range of size (Paulsen et al. 2018; Wang et al. 2021b). Under stress conditions, chromosomal DNA consistently generates substantial quantities of extrachromosomal DNA fragments (Bakhoum et al. 2018). The remarkable stability, mobility, and self-replication capacity of eccDNAs in animals make them exceptional vehicles for transmitting and amplifying signals between cells (Møller et al. 2018; Wang et al. 2021b; Zuo et al. 2022). EccDNAs possess transcriptional activity and can facilitate the expression of functional mRNAs and small regulatory RNAs (Paulsen et al. 2019; Wang et al. 2021b), thereby modulating gene expression. Here, we identified 71 distinct eccDNAs in Go-tomato plants, most of which overlapped with goji resequencing DNA fragments. Therefore, we speculate that the genetic material transferred during grafting is primarily composed of eccDNAs.

We also confirmed that eccDNAs were produced by the goji stock and that goji eccDNAs were transferred from the goji stock to the tomato scion, thus providing evidence for the ability of eccDNAs to traverse graft junctions. The transfer of goji eccDNAs primarily occurred across, or in close proximity to, the graft junctions, with negligible transfer observed in the upper leaves. This spatial pattern highlights apparent limitations in the transmission range of eccDNAs and provides insights into distance constraints for mobile genetic material transfer, which is potentially linked to the degree of scion cell differentiation. Among the 3780 annotated genes, 3 genes were associated with anthocyanin biosynthesis, 2 genes with cell division and differentiation, 2 genes with senescence, and 1 gene with plant growth and development.

EccDNAs appear to replicate independent of chromosomes; moreover, previous studies have proposed that these molecules undergo extrachromosomal replication through a rolling circle mechanism, as observed in several viral genomes and eukaryotes, such as mitochondrial DNA of the malaria parasite *Plasmodium falciparum* and the extrachromosomal amplification of ribosomal genes in the early oogenesis of amphibians (Cohen and Segal 2009; Ling et al. 2021). Our present study revealed that eccDNAs can be maintained in asexual offspring; this finding suggests that eccDNAs are replicable (otherwise the number of eccDNAs would reduce after mitotic division), and the conserved regions in eccDNAs (Fig. 4G and Supplemental Table S15) may be involved in the replication. Future studies should explore the role of the predicted consensus motifs in replication. Understanding the evolutionary and functional significance of conserved regions in eccDNAs can provide valuable insights into their maintenance and potential impact on asexual propagation, which strengthen our conclusion on the transfer of eccDNAs across graft junctions and shed light on the long-standing paradox of graft-derived genetic variation. This finding is consistent with our previous study on *Crucifer* grafting, whereby distinct small RNAs from donor parents were transmitted and maintained in asexual graft chimeras and subsequent sexual progenies (Chen et al. 2006; Liu et al. 2023; Zhu et al. 2007). We therefore postulate that stock-to-scion eccDNA transmission might also occur in *Crucifer* chimeras due to transmitted eccDNA-derived small RNAs. This possibility warrants further investigation to determine whether stock-to-scion eccDNAs transfer is a common mechanism in graft-induced genetic variation.

We observed unique transcripts originating from eccDNAs in G-AR transcriptomes, which provided insights into their possible role in enhancing the characteristics of scion tomato, such as cambium establishment, maintenance, and organ growth. This observation suggests that following the transfer of goji genetic material, these DNA fragments are transcribed and subsequently influence the transcriptome, ultimately leading to the acquisition of woody traits in Go-tomato. This hypothesis warrants further exploration.

In summary, we established an efficient inter-genera grafting model between distantly related woody and herbaceous plants by integrating in vitro culture and in vivo approaches. We confirmed the transfer of mobile DNA fragments between the stock and scion heterologous cells and within the homologous cells of Go-tomato regenerated roots or shoots in the form of replicable and expressible eccDNAs. These findings have high potential for the asexual production of crops, for example, by using a grafting system in the Solanaceae family plants, which considerably extended the growth cycle of the grafted plants in the field. Furthermore, the unique attributes of mobility and replicability of eccDNAs highlight their potential as vectors for horizontal DNA transfer through asexual grafting approaches. However, the molecular mechanisms underlying the changes in the growth characteristics of Go-tomato, including growth longevity and underground fruiting traits, need further investigations.

## Methods

### Plant materials

#### Scion preparation

Seeds from two tomato cultivars (*S. lycopersicum* cv. Condine Red, preserved in our laboratory through self-propagation, and *S. lycopersicum* cv. Zheyingfen No. 1, purchased from Zhejiang Yi Nong Seeds Ltd.) were used in this study. The seeds were soaked in water at 45 °C for 1 h, sterilized with 75% v/v ethanol for 30 s, and then treated with 10% v/v antiformin for 3 min. Subsequently, the seeds were soaked in sterile water and germinated at 25 °C in darkness. At the beginning of germination, seedlings were transferred to a hole tray filled with a moist matrix containing peat: perlite: vermiculite (60%:20%:20%, v/v) and cultured at 80% relative humidity at 26 °C/22°C under a 14-h light/10-h dark photoperiod with a light intensity of 120 µmol photons m^−2^ s^−1^; the cultured seeds were then used as in vivo grafting scions (Fig. 1B).

In vitro grafted scions were obtained by transferring the germinated seeds to ½ Murashige and Skoog (MS) medium and culturing them at 25 °C under a 12-h light/dark photoperiod with a light intensity of 100 µmol photons m^−2^ s^−1^.

#### Stock preparation

Goji (*L. ruthenicum*) stem segments with axillary buds were cultured in vitro in modified woody plant medium (WPM) and MS medium (1:1 volume ratio), which contained 30 g/L sucrose, 8 g/L agar, 0.4 mg/L isobutyl alcohol (IBA), and 0.05 mg/L naphthaleneacetic acid (NAA) (pH 5.8), at 22 °C with 14-h illumination at 120 µmol photons m^−2^ s^−1^. Following the growth of stems with axillary buds to 8 cm, they were cut 3 cm from the top and inoculated into the combined WPM and MS medium (1:1) containing 30 g/L sucrose, 8 g/L agar, and 0.05 mg/L IBA for root and shoot development. After 15 d, some of the in vitro grown goji seedlings were transferred to in vivo culture media for different durations to serve as in vivo stocks, while the remaining seedlings were used as in vitro stocks (Fig. 1A).

#### Grafting between goji and tomato

After approximately 15 d of growth of tomato seedlings, the portion above the cotyledons was cut and trimmed into a “V” shape; the remaining part was also cut into a “V” shape, and the two “Vs” were placed close together to form *t*omato *s*elf-grafted *p*lants (T-SPs). For goji, the top part was cut and trimmed into a “V” shape; the remaining part was cut longitudinally into a 0.8 cm split, and the two parts formed *g*oji *s*elf-grafted *p*lants (G-SPs). Additionally, the top part of goji was cut, and the leaves 2 cm below were collected and sleeved; a 0.8 cm split was made longitudinally so that the wedge-shaped lower end of the tomato could be inserted into the slit of goji to form *g*rafted *p*lants (GPs), with the sleeve adjusted accordingly. GPs were divided into two groups based on the presence or absence of regenerants, with the former designated as Go-tomato. T-SPs, G-SPs, GPs, and Go-tomato were grown for 3 d in airtight containers at 26 °C/22°C without illumination, followed by culture under 14-h illumination at 100–120 µmol photons m^−2^ s^−1^ with proper ventilation; the lid was partially opened to allow acclimation. Successful grafting was indicated by new leaf spread in the scion. Sterile tomato scions were also grafted onto goji stock in vitro.

#### Determination of lignin content in goji stock

Goji shoots at 10, 20, and 30 d of in vivo growth were selected, and a 1.5 cm section from the top of each shoot was cut. The leaves were removed, and stems were placed in a 15 mL centrifuge tube, dried overnight at 85 °C, ground into powder after reaching a constant weight, and then passed through a 40-mesh filter. Lignin was quantified using the acetyl bromide method; the absorbance of the sample was measured at 280 nm, and the lignin content was calculated based on a regression curve (Li 2006).

#### Microscopic observation

For stereomicroscope observations, hand-cut transverse sections of the grafted stem region were obtained. For light microscopy observation, the samples were fixed in a formaldehyde: acetic acid: ethanol fixative, washed twice with 70% v/v ethanol, and dehydrated in a graded ethanol series at room temperature (70%, 85%, 90%, and 95% v/v for 50 min, followed by 100% for 30 min, twice). The samples were infiltrated with a mixture of pure xylene and pure alcohol (1:1) for 30 min and subsequently infiltrated with pure xylene twice for 30 min. Shredded paraffin was slowly poured onto the samples containing xylene at room temperature to achieve a 50% v/v ratio of paraffin: xylene; the paraffin-embedded samples were then incubated overnight at 37℃. On the morning of the next day, the paraffin-immersed vessel was placed in an incubator at 60℃, the caps of the tubes were opened, and xylene was allowed to volatilize for 2 h. The samples were then transferred to fresh 100% paraffin in an incubator at 62 °C for 6 h, and the paraffin was replaced every 2 h. The pre-melted (60℃) pure paraffin was quickly poured into the embedding box. Once paraffin at the bottom of the embedding box solidified, the samples were moved rapidly into the embedding box, which was filled with paraffin. For light microscopy, polymerized samples were sectioned (8 μm thickness) with a paraffin slicing machine and mounted on glass slides. For light microscopy observation, the sections were stained with 0.5% w/v toluidine blue (pH 7.0).

For TEM observation, samples were fixed with 2.5% v/v glutaraldehyde at 4 °C overnight, washed three times with phosphate buffer (0.1 M, pH 7.0) for 15 min each, post-fixed with 1% w/v OsO_4_ in phosphate buffer for 1–2 h, and finally washed three times with phosphate buffer for 15 min each. The samples were dehydrated through a graded series of ethanol (30%, 50%, 70%, and 80%, v/v) for approximately 15 min each and then subjected to a graded series of acetone (90% and 95%, v/v) for approximately 15 min each. Finally, the samples were dehydrated twice with absolute acetone for 20 min each. The samples were then placed in a 1:1 mixture of absolute acetone and the final Spurr resin mixture for 1 h at room temperature, transferred to a 1:3 mixture of absolute acetone and the final Spurr resin mixture for 3 h, and then left to rest in the final Spurr resin mixture overnight. Subsequently, the samples were placed in an Eppendorf tube containing Spurr resin and heated at 70℃ for more than 9 h. The samples were then sectioned with an ultratome (Leica EM UC7), stained with uranyl acetate and alkaline lead citrate for 5–10 min each, and observed with a transmission electron microscope (Hitachi Model H-7650).

#### Determination of total-yield per plant

Fruits from T-SPs, GPs, and “Go-tomato” were harvested within 6 months after grafting, and 10 branches of fruits with consistent growth were selected for evaluation in each replicate.

#### Determination of physiological indices related to fruit quality

To assess fruit quality, three replicates (8 fruits each) of T-SPs and Go-tomato from the red ripening period sampled 5 months after grafting were ground into powder with liquid nitrogen. Total soluble solids were determined using a digital hand-held refractometer (Atago PAL-1, Japan) (Chu et al. 2018). Vitamin C content was measured by UV spectrophotometry (Li 2006). Anthocyanin content was assessed using the pH differential method as reported earlier (Correa-Betanzo et al. 2014).

#### Genome resequencing

Five- to 8-cm-long root tissues from Go-tomato root tips were collected and stored at − 80℃ until DNA isolation. Six biological replicates were performed, with each replicate containing root tissues from three grafts. Genomic DNA was extracted using a modified CTAB method (Huang et al. 2016). The sequencing library (30×) was prepared according to the standard protocol of Illumina, and sequencing was conducted by BGI Genomics Co., Ltd. Clean data were aligned to the tomato CR (*S. lycopersicum* cv. Condine Red) genome by using Burrows–Wheeler Aligner (BWA) software (Li 2009). The Genome Analysis Toolkit was used for base recalibration and realignment near insertion or deletion regions (Li 2009). SAMtools (http://samtools.sourceforge.net) was used to estimate reference genome coverage of single nucleotide polymorphisms and insertion-deletion variants for identification and analysis. The files of samples in fq.gz format for the Go-tomato data were compared with the tomato genome by BWA mem (Li 2009), and the reads aligned to tomato were removed to obtain the data shown in Supplemental Table S2. The remaining reads from six samples of Go-tomato were subsequently mapped to the goji genome to obtain sequences with depth intervals (Supplemental Tables S3 and S4).

#### PCR

Total cellular DNA was amplified in an Eppendorf thermal cycler by using 2× Rapid Taq Master Mix (Vazyme) and specific primer pairs (Supplemental Table S17) designed based on the selected sequences. The standard PCR program was as follows: 3 min at 95 °C, followed by 30 cycles of 94 °C for 15 s, 55 °C for 15 s, and 72 °C for 10 s and a final extension at 72 °C for 10 min. PCR validation of eccDNAs was performed using the method described by Zhuang et al. (2024).

#### SRAP analysis

The first five cycles were run at 94 °C for 1 min, 37 °C for 1 min, and 72 °C for 10 s for denaturing, annealing, and extension, respectively. The annealing temperature was then increased to 54 °C for an additional 40 cycles (Li et al. 2001). Amplicons were separated on denaturing acrylamide gels and detected by autoradiography. Primers used are listed in Supplemental Table S17.

#### Whole-genome sequencing

Genomic DNA was isolated from tomato CR and 5- to 8-cm tissues from the root tip of Go-tomato. Tissues were immediately frozen in liquid nitrogen before extraction. Total genomic DNA was extracted using the CTAB method (Huang et al. 2016) and physically sheared to 10- to 20-kb fragments to construct sequencing libraries following PacBio HiFi circular consensus sequencing. We used Hifiasm (Cheng et al. 2021) to assemble the two genomes with -k 63 as the genome setting, and the 12 chromosome sequences were connected by Ragtag (Alonge et al. 2022). The quality and completeness of genome assemblies were evaluated using BUSCO v5.1 (Seppey et al. 2019) based on the embryophyta_odb10. To identify structural variants between the tomato and Go-tomato genomes, variants such as deletions (Aloni et al. 2015), insertions (INSs), duplications (DUPs), inversions (INVs), and translocations (TRAs) were detected using SyRI (Goel et al. 2019).

#### Mitochondrial genome sequencing

Roots of goji and Go-tomato were washed with sterile water. Root tissues at approximately 5–8 cm above the root tip from similar locations were sampled, dried with nonwoven fabrics, and then frozen in liquid nitrogen. Purified DNA was used to construct sequencing libraries with an insert size of 450 bp in accordance with the Illumina sequencing protocol. Fragments of an average size of 10 kb were selected, and one single-molecule real-time (SMRT) sequencing cell was sequenced on the PacBio RSII platform by using P6-C4 reagents (Pacific Biosciences, USA). The Illumina raw data output was checked individually in FastQC version 0.11.5 (http://www.bioinformatics.babraham.ac.uk/projects/fastqc) and then trimmed with Trimmomatic version 0.32 (Bolger et al. 2014). By using a list of published tomato mitochondrial genome as a reference, pair-end reads that aligned at least once to the reference were selected in BWA version 0.7.17 (Li 2009). These reads were then assembled *de novo* using SPAdes 3.9.0 (Bankevich et al. 2012) with the “-k 21,33,55,77,99,127 -- careful” option. PacBio raw reads were assembled individually using MECAT version 1.2 and CANU version 1.5 (Koren et al. 2017; Xiao et al. 2017). By aligning the contigs from SPAdes, each mitochondrial genome was assembled into two or three contigs. PCR validation was performed to obtain a master circle mitochondrial genome. All reads were realigned to the master circle mitochondrial genome to detect any mismatches or indels. Pilon version 1.23 (Bankevich et al. 2012) was used to polish the sequences.

Functional genes of the mitochondrial genome were annotated by BLAST against a local database derived from the published tomato mitochondrial genome in GenBank, and tRNAs were identified using tRNAscan-SE 2.0 (Lowe and Chan 2016). The open reading frames and genes were modified and predicted with the NCBI Open Reading Frame Finder (https://www.ncbi.nlm.nih.gov/orffinder/) and then annotated by BLASTX queries against the non-redundant NCBI database. The mitochondrial genome circular map was drawn by OGDRAW (Lohse et al. 2013). To align the starting sites of all genomes, one of the genomes was used as a reference, and the starting sequence of all other genomes was adjusted. Sequence alignment was then obtained by Mauve (version 2.3.1) (Darling et al. 2004).

#### Chloroplast-genome differential sequence alignment

Total DNA was extracted from goji, tomato, and Go-tomato root tissues for PCR to differentiate between the chloroplast genomes of goji and tomato. SnapGene was used to select primers based on the tomato chloroplast genome (https://www.ncbi.nlm.nih.gov/search/all/?term=NC_007898.3) and the goji chloroplast genome (https://www.ncbi.nlm.nih.gov/search/all/?term=NC_039651.1&source=taxonomy).

Differences in sequence sizes between the chloroplast genomes of goji and tomato were noted for the same primers. To investigate whether the goji chloroplast genome fragments were transferred into tomato cells, synthetic oligonucleotides were used as primers (Supplemental Table S17) for PCR, following the same standards as mentioned above.

#### EccDNA sequencing

Ungrafted goji plants (Goji), self-grafted goji stocks (S-goji), self-grafted tomato scions (S-tomato), grafted tomato scions (G-tomato), and 5- to 8-cm regenerated adventitious root tissues upward from the root tip of Go-tomato (G-AR) were collected for the isolation and sequencing of DNA. Each sample comprised tissues from three plants. Stem segments from goji and S-goji were sampled at 8 cm from the bottom; the samples were approximately 2 cm in length and obtained at 10 d after grafting. Stem segments from S-tomato and G-tomato were sampled at 0.8 cm above the graft junction at 10 d after grafting. Asexual progeny from tomato and Go-tomato were sampled as 5-cm stem segments from the bottom at 1 month after cutting.

Purification of eccDNAs was optimized based on the Circle-seq eccDNA method (Deng et al. 2022; Huang et al. 2022). Genomic DNA was extracted using Qiagen kits, and total DNA was subjected to alkaline pH treatment to separate chromosomal DNA, lipids, and proteins through rapid DNA denaturing–renaturing, followed by column chromatography on an ion-exchange membrane column (Plasmid Mini AX; A&A Biotechnology). The remaining linear DNA was removed by an exonuclease (plasmid-safe ATP-dependent DNase, Epicentre) together with the rare-cutting endonuclease *Mss*I that digests mitochondrial circular DNA (16 kb) and creates additional accessible DNA ends for exonuclease action.

EccDNA-enriched samples were used as templates for phi29 polymerase reactions (REPLI-g Midi Kit) to amplify DNA. The amplified circular DNA was cleaned (AMPure XP beads) and sheared by sonication (Bioruptor) to obtain fragments with an average size of 200–300 bp. Libraries for next-generation sequencing were prepared using the NEBNext Ultra DNA Library Kit for Illumina in accordance with the manufacturer’s protocol (New England Biolabs) and sequenced on an Illumina Novaseq 6000 platform using PE150.

Trimmomatic version 0.32 (Bolger et al. 2014) was used to remove adapters and low-quality reads. Clean reads were then aligned to the goji reference genome by using BWA version 0.7.17 (Li 2009). Circular DNA was detected by Circle Map to obtain data for the seven samples. To determine whether eccDNA is produced in goji directly through grafting or independently, BWA version 0.7.17 (Li 2009) was used to compare the grafted rootstock data to the goji control data. To establish whether goji eccDNA is transferred from the goji rootstock to the tomato scion, the grafted tomato scion data were used to identify differential sequences, which were then compared with the grafted goji rootstock data to find common eccDNA sources by BWA version 0.7.17 (Li 2009). To verify whether goji eccDNA can be maintained and transmitted in asexual progeny, eccDNA data from the asexual progeny of Go-tomato were compared with eccDNA data from the asexual progeny of tomato to obtain differential sequences; these sequences were then mapped to goji-specific sequences to identify goji-specific eccDNA by using BWA version 0.7.17 (Li 2009). To investigate the presence of conserved sequences in eccDNAs maintained in asexual offspring, the sequence features of eccDNAs were examined using MEME (https://meme-suite.org/meme/opal-jobs/appMEME_5.5.717255067415341578431199/meme.html#sites_sec).

#### Transcriptome analysis

T-SPs and Go-tomato were harvested at 50 and 80 d after grafting, respectively. Root tissues at approximately 5–8 cm above the root tip from similar locations were sampled. Three biological replicates were prepared for each tissue type, with each replicate comprising root tissues from three grafts or T-SPs. Total RNA was extracted using a standard method (Tan et al. 2009). DNA libraries were prepared with the NEBNext^®^ Ultra™ RNA Library Prep Kit for Illumina^®^ in accordance with the manufacturer’s protocol and sequenced on an Illumina NovaSeq 6000 platform (Illumina, USA). Data preprocessing included filtering raw data to remove reads with adapters, reads containing Ns (undetermined base information), and low-quality reads (bases with Phred quality score ≤ 20 accounting for more than 50% of the total read length). Data quality was assessed with fastp (version 0.19.7) (Chen et al. 2018). Clean reads were mapped to the genome assembly using HISAT2 (Gill et al. 2022). SAM files were converted to BAM format using SAMtools version 1.4.1 (http://samtools.sourceforge.net). StringTie (Gill et al. 2022) was used to assemble new transcripts, which were then annotated using Pfam, SUPERFAMILY, Gene Ontology (GO), Kyoto Encyclopedia of Genes and Genomes (KEGG), and other databases. Gene expression was quantified using FeatureCounts in Subreads (Liao et al. 2014), and DESeq2 (Love et al. 2014) was used for differential expression analysis. GO functional and KEGG pathway enrichment analyses of DEGs were performed with ClusterProfiler software.

## Supplementary Information

Supplementary Material 1: Fig. S1. Establishment of a grafting system between distant plant species and organogenesis of tomato stem cells (related to Fig. 1). A, Cell wall thickness in goji stems after grafting. Differences between mean values were analyzed by Fisher’s exact test (*P < 0.05;*n* = 3). B, Survival rate of grafted plants after grafting with goji at the indicated days. Differences between mean values were analyzed by Fisher’s exact test (****P < 0.0001). C, Shoot growth at 80 d after grafting. T-SPs, tomato self-grafted plants; Go-tomato, grafted plants with regenerants at 80 d after grafting; GPs, grafted plants. Scale bar = 10 cm. D, Changes in physiological indices after grafting. Top: Plant height at 10 weeks after grafting. Bottom: Chlorophyll content of opposite leaves of the third, fifth, and seventh inflorescences after grafting as determined by spectrophotometry. T-SPs, tomato self-grafted plants; Go-tomato, grafted plants with regenerants; GPs, grafted plants. Differences between mean values were analyzed by Fisher’s exact test (*P < 0. 1, ***P < 0.001; *n* = 9). E, Regenerated buds from Go-tomato plants. F, Go-tomato production in Qinghai, China, in December 2020. G, Yield and fruit quality after grafting. T-SPs, tomato self-grafted plants; Go-tomato, plant with regenerants; GPs, grafted plants. Left to right: Total yield per plant within 6 months after grafting, and anthocyanin content, vitamin C content, and total soluble solids content at 5 months after grafting. Differences between mean values were analyzed by Fisher’s exact test (***P< 0.001; *n* = 5). Fig. S2. Resequencing of “Go-tomato” (related to Fig. 3). A, Analysis pipeline for whole-genome resequencing. B, Median read density of Go-tomato_PR_1_3 and Go-tomato_LR_1–3 samplesmapped to the goji genome. Window length = 100 kb. Median read density represents count per window length. Fig. S3. Goji DNA fragments transferred to tomato by grafting (related to Fig. 3). A, Mapping results of the goji genome. “Chr” represents the chromosome to which the resequencing data are mapped; length represents the length of sequences mapped to the chromosome; Go-tomato_1 represents the communal sequence of Go-tomato_PR_1 and Go-tomato_LR_1; Go-tomato_2 represents the communal sequence of Go-tomato_PR_2 and Go-tomato_LR_2; Go-tomato_3 represents the communal sequence of Go-tomato_PR_3 and Go-tomato_LR_3; Go-tomato_PR represents the communal sequence of Go-tomato_PR_1, Go-tomato_PR_2, and Go-tomato_PR_3; Go-tomato_LR represents the communal sequence of Go-tomato_LR_1, Go-tomato_LR_2, and Go-tomato_LR_3. B, Horizontal gene transfer (HGT) frequency of 24 adventitious roots from 24 unique grafted plant samples. C, Amplification of HGT-1 (1105 bp) and HGT-2 (1123 bp) in the primary roots and lateral roots of G-AR. PR: Primary roots; LR: Lateral roots. Sl_1, 2, 3; G-AR_PR_4_1, 2, 3; and G-AR_LR_4_1_1, 2, 3: Three technical replicates. G-AR_LR_4_1, G-AR_LR_4_2, and G-AR_LR_4_3 represent three biological replicates from the same G-AR. D, Amplification of HGT-1 (1105 bp) in the sampling position of Go-tomato. 5 and 6 represent two individual Go-tomato plants. A–D represent different sampling positions. A, red box; B, blue box; C, yellow box; D, green box; *Sl*, tomato; *Lr*, goji. Fig. S4. Source of the transferred DNA fragments from goji (related to Fig. 3). A, Mitogenome map ofgoji. B, Mitogenome map of Go-tomato. C, Genomic collinearity analysis of Go-tomato, goji, and tomato. D, PCR amplification of Chloroplast-1 and Chloroplast-2 in tomato (*Sl*), goji (*Lr*), and Go-tomato roots. Go-tomato_7 to Go-tomato_20 represent different individuals. *Sl + Lr* represents a 1:1 DNA mixture of tomato (*Sl*) and goji (*Lr*) used as controls. Fig. S5. Genome size determination in Go-tomato by flow cytometry (related to Fig. 3). A–H, Flow cytometry analysis of different plants. A, Tomato self-grafted plant leaves; B, Grafted plant leaves; C, “Go-tomato” leaves, D, Goji leaves, E, Tomato self-grafted plant root system, F, Grafted plant root system, G, Go-tomato primary root, and H, “Go-tomato” lateral root. Fig. S6. Detection of eccDNAs in different tissues (related to Fig. 4). A, Venn diagrams displaying overlaps of eccDNA populations detected between two biological replicates from G-AR. B, Overall chromosomal distribution of 99 eccDNAs across the goji genome in G-AR. Peak IDs represent the ID number of eccDNAs in this analysis. Dark blue: Position of each eccDNA; light blue: length of each eccDNA. C, PCR-based identification of eccDNAs chr04_141232335_141234901 (upper) and chr05_74293185_74293747 (lower) across G-AR. D, Venn diagrams displaying overlap of eccDNA populations between S-goji and Goji. E, Location of Goji and S-goji eccDNAs across the 12 goji chromosomes. Red line: S-goji. Blue line: Goji. Fig. S7. Grafting-induced changes in gene expression in the tomato regenerated adventitious root system (related to Fig. 5). A, Volcano plots of DEGs. The two upper panels show DEGs between the regenerated primary roots of Go-tomato and tomato self-grafted plants (T-SPs) at 50 d after grafting (DEG cluster 1) and between the regenerated primary roots of Go-tomato and T-SPs at 80 d after grafting (DEG cluster 2). The two lower panels show DEGs between the regenerated secondary roots of Go-tomato and T-SPs at 50 d after grafting (DEG cluster 3) and between the secondary roots of Go-tomato and T-SPs at 80 d after grafting (DEG cluster 4). B, GO enrichment analysis of two different combinations. The two panels indicate GO enrichment analysis of DEG clusters 1 and 3 and DEG clusters 2 and 4, respectively. C, KEGG pathway enrichment scatterplot. The two upper plots show KEGG pathway enrichment analysis of b and c in Panel A; the lower lane indicates KEGG pathway enrichment analysis of d in Panel A. D, PCR-based amplification of HGT-1 (1105 bp) in the regenerated primary roots of Go-tomato and T-SPs at 50 d after grafting.

Supplementary Material 2:  Table S1. Resequencing data filtering statistics.

Supplementary Material 3:  Table S2. Whole-genome resequencing and alignment.

Supplementary Material 4:  Table S3. Mapping of Go-tomato samples to the goji genome after excluding data aligned to the tomato genome.

Supplementary Material 5:  Table S4. Location of Go-tomato samples mapped to the goji genome.

Supplementary Material 6:  Table S5. Sequence information matching with organelles for Go-tomato samples.

Supplementary Material 7:  Table S6. Statistics of genome-wide assembly results.

Supplementary Material 8:  Table S7. EccDNA data-filtering statistics.

Supplementary Material 9:  Table S8. Mapping rate of eccDNA reads to the goji genome.

Supplementary Material 10: Table S9. Detection of eccDNAs.

Supplementary Material 11:  Table S10. Comparison of eccDNA positions between G-AR-1 and G-AR-2.

Supplementary Material 12:  Table S11. EccDNA coordinated file of G-AR-2.

Supplementary Material 13:  Table S12. Consensus sequence of six samples mapped to filter eccDNAs detected in G-AR-2.

Supplementary Material 14:  Table S13. EccDNA annotation results for G-AR-2.

Supplementary Material 15:  Table S14. Statistics of grafted goji stock (S-goji) eccDNAs transferred to tomato scions (G-tomato).

Supplementary Material 16:  Table S15. Motif consensus of 13 eccDNAs maintained in asexual offspring.

Supplementary Material 17:  Table S16. Unique transcripts of G-AR-2 compared with eccDNAs.

Supplementary Material 18:  Table S17. Primer sequences for PCR.

## Data Availability

All data used for this article are publicly accessible listed under “Gene and Accession Numbers”.

## Abbreviations

GPs: grafted plants
G-SPs: goji self-grafted plants
G-tomato: grafted tomato scion
Goji: Ungrafted goji plants
S-goji: self-grafted goji stock
S-tomato: self-grafted tomato scion
T-SPs: tomato self-grafted plants
G-AR: adventitious root of Go-tomato
