# Supplementary figures and images for: Horizontal transfer of plasmid-like extrachromosomal circular DNAs across graft junctions in Solanaceae
Source: Mol Hortic. 2024 Nov 20;4:41. doi: 10.1186/s43897-024-00124-0 (PMC11577957; doi:10.1186/s43897-024-00124-0)

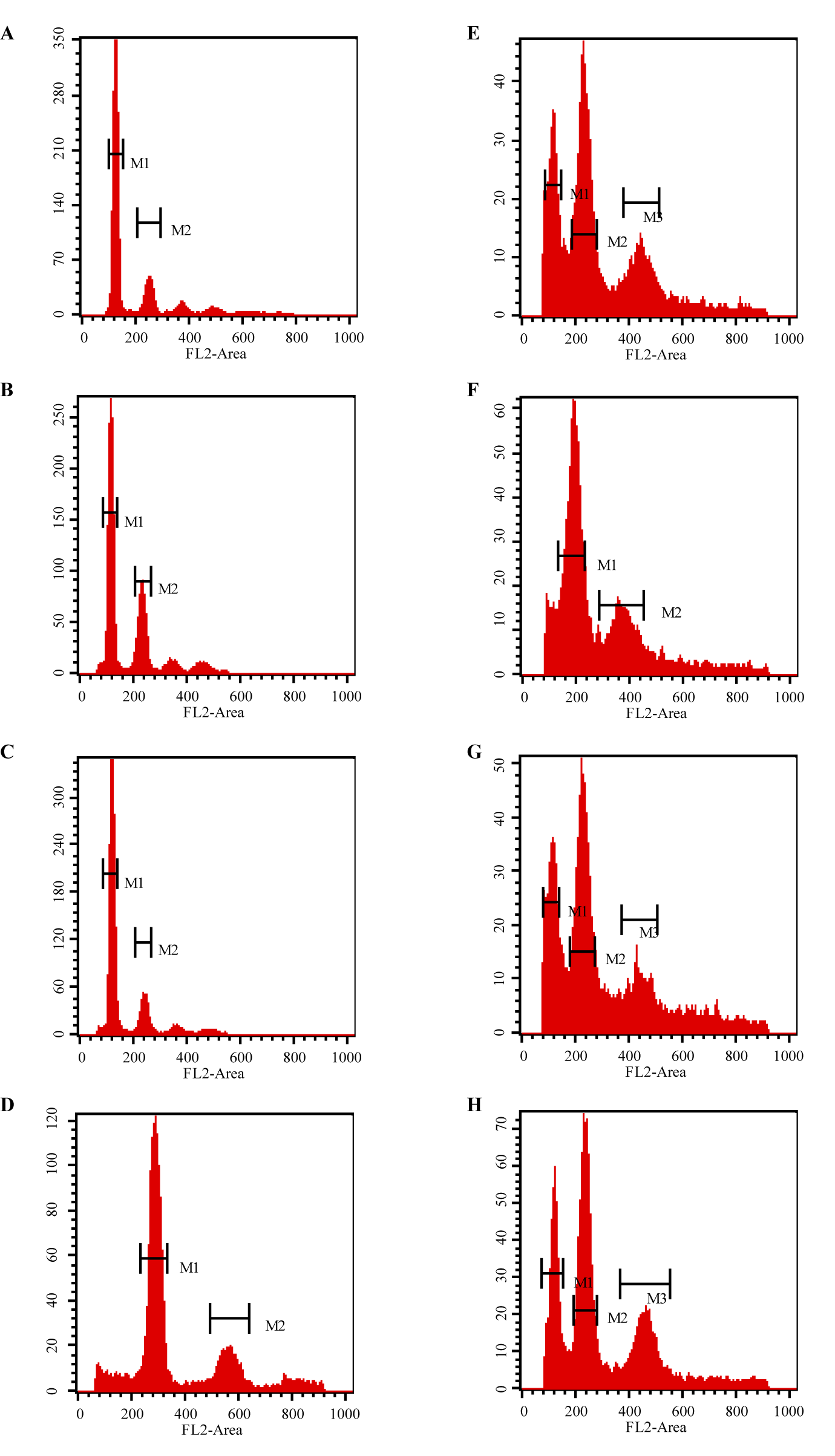

Supplement: Supplementary file 1 — Supplementary Material 1: Fig. S1. Establishment of a grafting system between distant plant species and organogenesis of tomato stem cells (related to Fig. 1). A, Cell wall thickness in goji stems after grafting. Differences between mean values were analyzed by Fisher’s exact test (*P < 0.05;n = 3). B, Survival rate of grafted plants after grafting with goji at the indicated days. Differences between mean values were analyzed by Fisher’s exact test (****P < 0.0001). C, Shoot growth at 80 d after grafting. T-SPs, tomato self-grafted plants; Go-tomato, grafted plants with regenerants at 80 d after grafting; GPs, grafted plants. Scale bar = 10 cm. D, Changes in physiological indices after grafting. Top: Plant height at 10 weeks after grafting. Bottom: Chlorophyll content of opposite leaves of the third, fifth, and seventh inflorescences after grafting as determined by spectrophotometry. T-SPs, tomato self-grafted plants; Go-tomato, grafted plants with regenerants; GPs, grafted plants. Differences between mean values were analyzed by Fisher’s exact test (*P < 0. 1, ***P < 0.001; n = 9). E, Regenerated buds from Go-tomato plants. F, Go-tomato production in Qinghai, China, in December 2020. G, Yield and fruit quality after grafting. T-SPs, tomato self-grafted plants; Go-tomato, plant with regenerants; GPs, grafted plants. Left to right: Total yield per plant within 6 months after grafting, and anthocyanin content, vitamin C content, and total soluble solids content at 5 months after grafting. Differences between mean values were analyzed by Fisher’s exact test (***P< 0.001; n = 5). Fig. S2. Resequencing of “Go-tomato” (related to Fig. 3). A, Analysis pipeline for whole-genome resequencing. B, Median read density of Go-tomato_PR_1_3 and Go-tomato_LR_1–3 samplesmapped to the goji genome. Window length = 100 kb. Median read density represents count per window length. Fig. S3. Goji DNA fragments transferred to tomato by grafting (related to Fig. 3). A, Mapping results of the [file 43897_2024_124_MOESM1_ESM.zip › 43897_2024_124_Fig10_Print.jpeg]

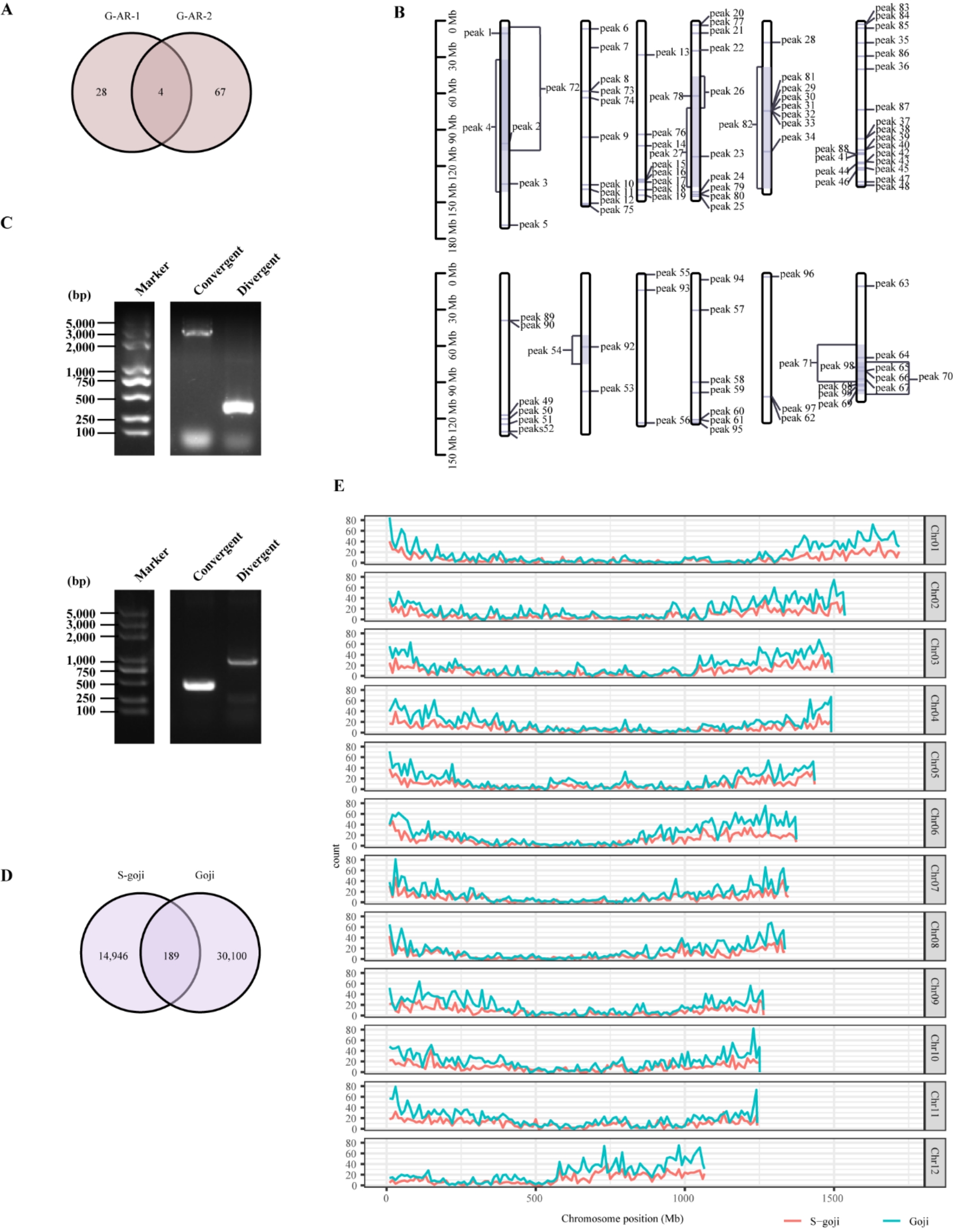

Supplement: Supplementary file 1 — Supplementary Material 1: Fig. S1. Establishment of a grafting system between distant plant species and organogenesis of tomato stem cells (related to Fig. 1). A, Cell wall thickness in goji stems after grafting. Differences between mean values were analyzed by Fisher’s exact test (*P < 0.05;n = 3). B, Survival rate of grafted plants after grafting with goji at the indicated days. Differences between mean values were analyzed by Fisher’s exact test (****P < 0.0001). C, Shoot growth at 80 d after grafting. T-SPs, tomato self-grafted plants; Go-tomato, grafted plants with regenerants at 80 d after grafting; GPs, grafted plants. Scale bar = 10 cm. D, Changes in physiological indices after grafting. Top: Plant height at 10 weeks after grafting. Bottom: Chlorophyll content of opposite leaves of the third, fifth, and seventh inflorescences after grafting as determined by spectrophotometry. T-SPs, tomato self-grafted plants; Go-tomato, grafted plants with regenerants; GPs, grafted plants. Differences between mean values were analyzed by Fisher’s exact test (*P < 0. 1, ***P < 0.001; n = 9). E, Regenerated buds from Go-tomato plants. F, Go-tomato production in Qinghai, China, in December 2020. G, Yield and fruit quality after grafting. T-SPs, tomato self-grafted plants; Go-tomato, plant with regenerants; GPs, grafted plants. Left to right: Total yield per plant within 6 months after grafting, and anthocyanin content, vitamin C content, and total soluble solids content at 5 months after grafting. Differences between mean values were analyzed by Fisher’s exact test (***P< 0.001; n = 5). Fig. S2. Resequencing of “Go-tomato” (related to Fig. 3). A, Analysis pipeline for whole-genome resequencing. B, Median read density of Go-tomato_PR_1_3 and Go-tomato_LR_1–3 samplesmapped to the goji genome. Window length = 100 kb. Median read density represents count per window length. Fig. S3. Goji DNA fragments transferred to tomato by grafting (related to Fig. 3). A, Mapping results of the [file 43897_2024_124_MOESM1_ESM.zip › 43897_2024_124_Fig11_Print.jpeg]

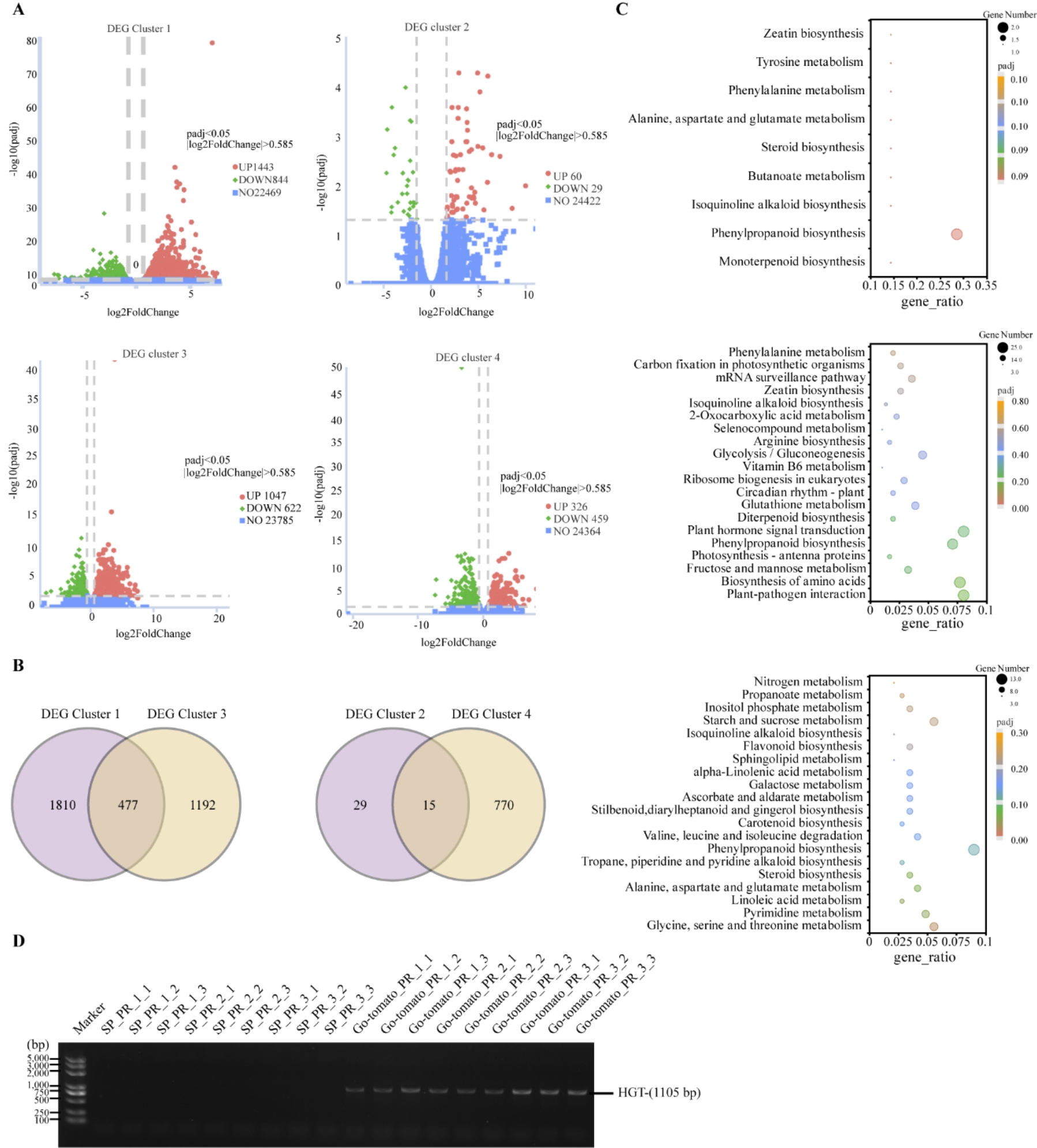

Supplement: Supplementary file 1 — Supplementary Material 1: Fig. S1. Establishment of a grafting system between distant plant species and organogenesis of tomato stem cells (related to Fig. 1). A, Cell wall thickness in goji stems after grafting. Differences between mean values were analyzed by Fisher’s exact test (*P < 0.05;n = 3). B, Survival rate of grafted plants after grafting with goji at the indicated days. Differences between mean values were analyzed by Fisher’s exact test (****P < 0.0001). C, Shoot growth at 80 d after grafting. T-SPs, tomato self-grafted plants; Go-tomato, grafted plants with regenerants at 80 d after grafting; GPs, grafted plants. Scale bar = 10 cm. D, Changes in physiological indices after grafting. Top: Plant height at 10 weeks after grafting. Bottom: Chlorophyll content of opposite leaves of the third, fifth, and seventh inflorescences after grafting as determined by spectrophotometry. T-SPs, tomato self-grafted plants; Go-tomato, grafted plants with regenerants; GPs, grafted plants. Differences between mean values were analyzed by Fisher’s exact test (*P < 0. 1, ***P < 0.001; n = 9). E, Regenerated buds from Go-tomato plants. F, Go-tomato production in Qinghai, China, in December 2020. G, Yield and fruit quality after grafting. T-SPs, tomato self-grafted plants; Go-tomato, plant with regenerants; GPs, grafted plants. Left to right: Total yield per plant within 6 months after grafting, and anthocyanin content, vitamin C content, and total soluble solids content at 5 months after grafting. Differences between mean values were analyzed by Fisher’s exact test (***P< 0.001; n = 5). Fig. S2. Resequencing of “Go-tomato” (related to Fig. 3). A, Analysis pipeline for whole-genome resequencing. B, Median read density of Go-tomato_PR_1_3 and Go-tomato_LR_1–3 samplesmapped to the goji genome. Window length = 100 kb. Median read density represents count per window length. Fig. S3. Goji DNA fragments transferred to tomato by grafting (related to Fig. 3). A, Mapping results of the [file 43897_2024_124_MOESM1_ESM.zip › 43897_2024_124_Fig12_Print.jpeg]

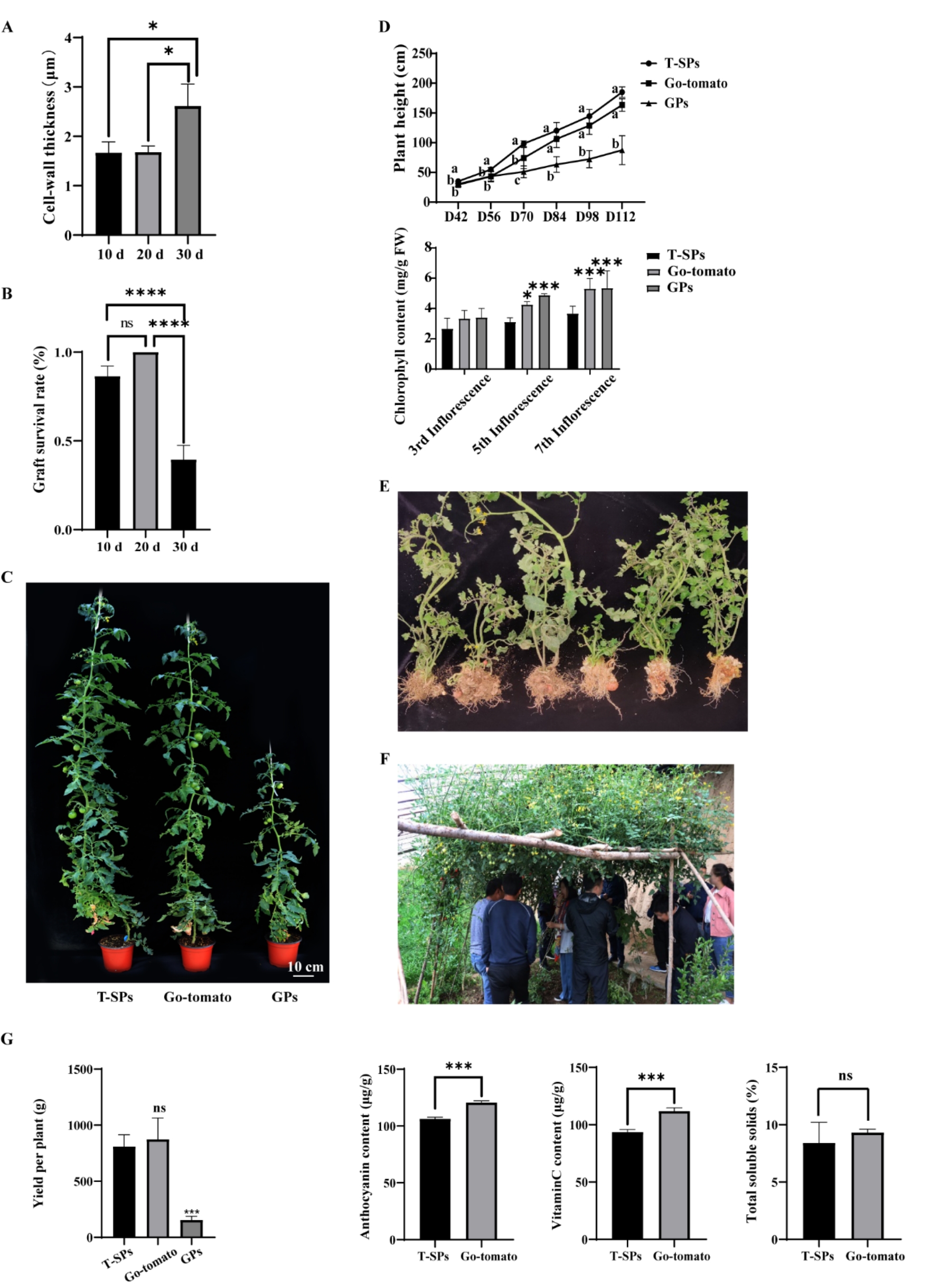

Supplement: Supplementary file 1 — Supplementary Material 1: Fig. S1. Establishment of a grafting system between distant plant species and organogenesis of tomato stem cells (related to Fig. 1). A, Cell wall thickness in goji stems after grafting. Differences between mean values were analyzed by Fisher’s exact test (*P < 0.05;n = 3). B, Survival rate of grafted plants after grafting with goji at the indicated days. Differences between mean values were analyzed by Fisher’s exact test (****P < 0.0001). C, Shoot growth at 80 d after grafting. T-SPs, tomato self-grafted plants; Go-tomato, grafted plants with regenerants at 80 d after grafting; GPs, grafted plants. Scale bar = 10 cm. D, Changes in physiological indices after grafting. Top: Plant height at 10 weeks after grafting. Bottom: Chlorophyll content of opposite leaves of the third, fifth, and seventh inflorescences after grafting as determined by spectrophotometry. T-SPs, tomato self-grafted plants; Go-tomato, grafted plants with regenerants; GPs, grafted plants. Differences between mean values were analyzed by Fisher’s exact test (*P < 0. 1, ***P < 0.001; n = 9). E, Regenerated buds from Go-tomato plants. F, Go-tomato production in Qinghai, China, in December 2020. G, Yield and fruit quality after grafting. T-SPs, tomato self-grafted plants; Go-tomato, plant with regenerants; GPs, grafted plants. Left to right: Total yield per plant within 6 months after grafting, and anthocyanin content, vitamin C content, and total soluble solids content at 5 months after grafting. Differences between mean values were analyzed by Fisher’s exact test (***P< 0.001; n = 5). Fig. S2. Resequencing of “Go-tomato” (related to Fig. 3). A, Analysis pipeline for whole-genome resequencing. B, Median read density of Go-tomato_PR_1_3 and Go-tomato_LR_1–3 samplesmapped to the goji genome. Window length = 100 kb. Median read density represents count per window length. Fig. S3. Goji DNA fragments transferred to tomato by grafting (related to Fig. 3). A, Mapping results of the [file 43897_2024_124_MOESM1_ESM.zip › 43897_2024_124_Fig6_Print.jpeg]

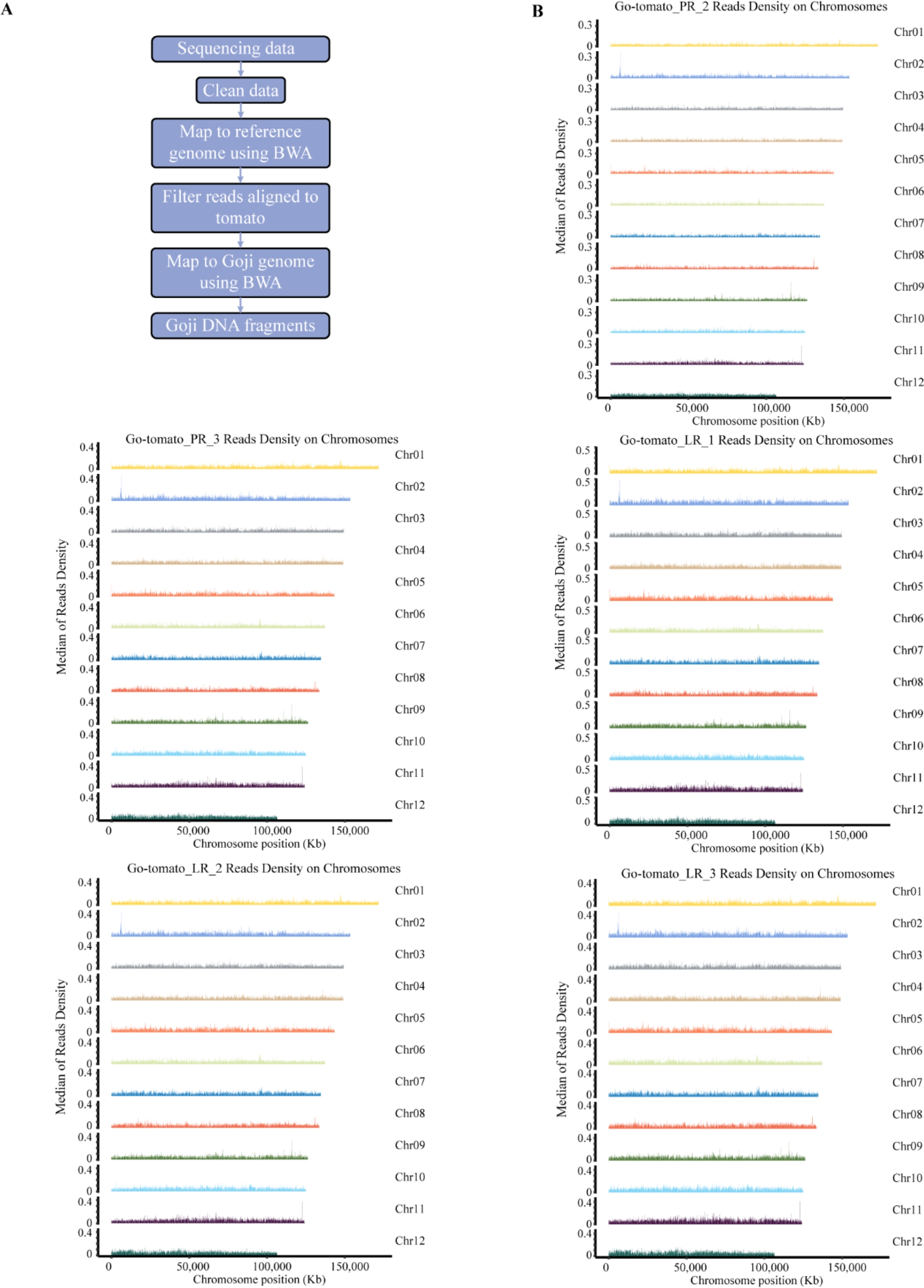

Supplement: Supplementary file 1 — Supplementary Material 1: Fig. S1. Establishment of a grafting system between distant plant species and organogenesis of tomato stem cells (related to Fig. 1). A, Cell wall thickness in goji stems after grafting. Differences between mean values were analyzed by Fisher’s exact test (*P < 0.05;n = 3). B, Survival rate of grafted plants after grafting with goji at the indicated days. Differences between mean values were analyzed by Fisher’s exact test (****P < 0.0001). C, Shoot growth at 80 d after grafting. T-SPs, tomato self-grafted plants; Go-tomato, grafted plants with regenerants at 80 d after grafting; GPs, grafted plants. Scale bar = 10 cm. D, Changes in physiological indices after grafting. Top: Plant height at 10 weeks after grafting. Bottom: Chlorophyll content of opposite leaves of the third, fifth, and seventh inflorescences after grafting as determined by spectrophotometry. T-SPs, tomato self-grafted plants; Go-tomato, grafted plants with regenerants; GPs, grafted plants. Differences between mean values were analyzed by Fisher’s exact test (*P < 0. 1, ***P < 0.001; n = 9). E, Regenerated buds from Go-tomato plants. F, Go-tomato production in Qinghai, China, in December 2020. G, Yield and fruit quality after grafting. T-SPs, tomato self-grafted plants; Go-tomato, plant with regenerants; GPs, grafted plants. Left to right: Total yield per plant within 6 months after grafting, and anthocyanin content, vitamin C content, and total soluble solids content at 5 months after grafting. Differences between mean values were analyzed by Fisher’s exact test (***P< 0.001; n = 5). Fig. S2. Resequencing of “Go-tomato” (related to Fig. 3). A, Analysis pipeline for whole-genome resequencing. B, Median read density of Go-tomato_PR_1_3 and Go-tomato_LR_1–3 samplesmapped to the goji genome. Window length = 100 kb. Median read density represents count per window length. Fig. S3. Goji DNA fragments transferred to tomato by grafting (related to Fig. 3). A, Mapping results of the [file 43897_2024_124_MOESM1_ESM.zip › 43897_2024_124_Fig7_Print.jpeg]

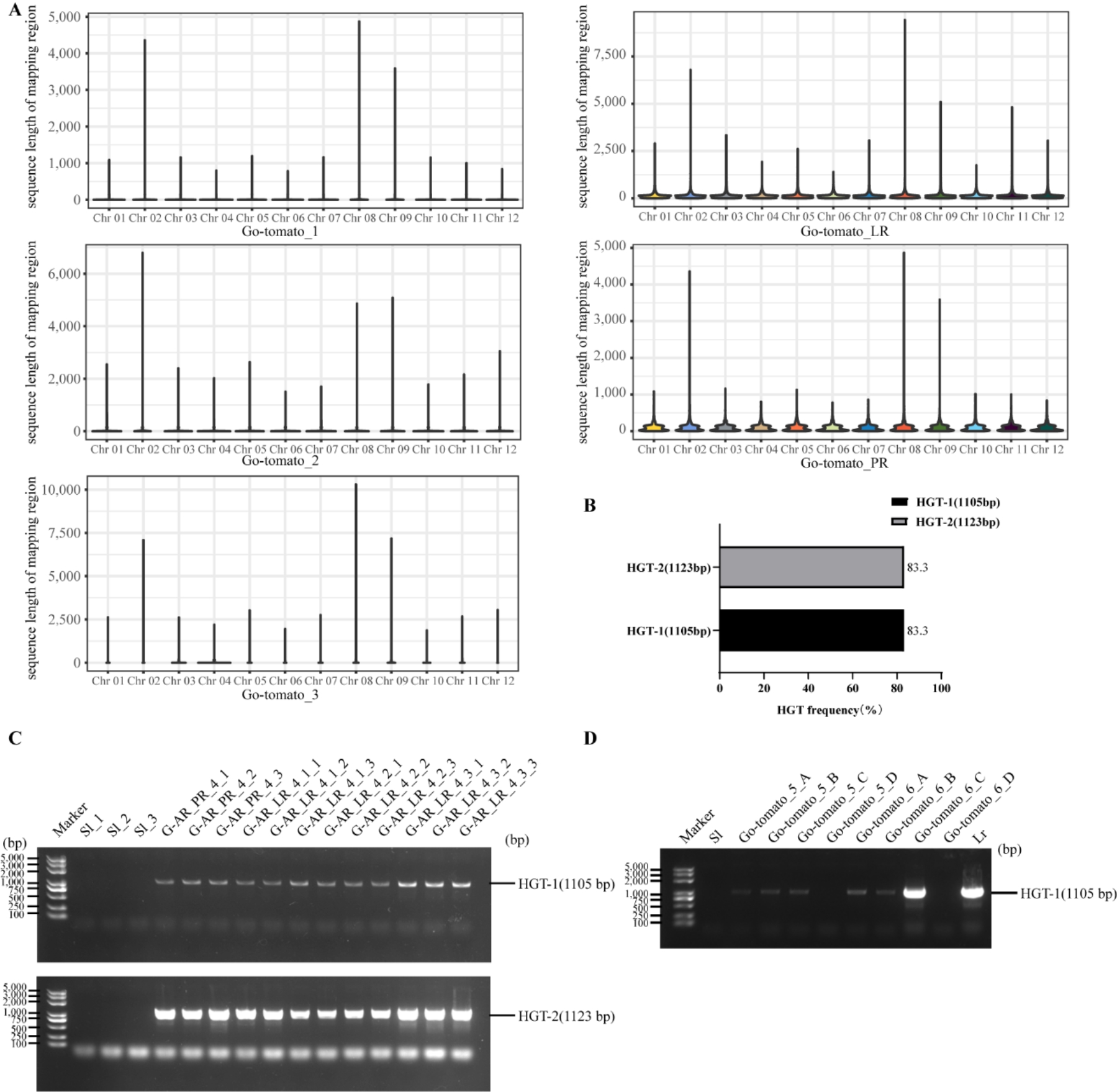

Supplement: Supplementary file 1 — Supplementary Material 1: Fig. S1. Establishment of a grafting system between distant plant species and organogenesis of tomato stem cells (related to Fig. 1). A, Cell wall thickness in goji stems after grafting. Differences between mean values were analyzed by Fisher’s exact test (*P < 0.05;n = 3). B, Survival rate of grafted plants after grafting with goji at the indicated days. Differences between mean values were analyzed by Fisher’s exact test (****P < 0.0001). C, Shoot growth at 80 d after grafting. T-SPs, tomato self-grafted plants; Go-tomato, grafted plants with regenerants at 80 d after grafting; GPs, grafted plants. Scale bar = 10 cm. D, Changes in physiological indices after grafting. Top: Plant height at 10 weeks after grafting. Bottom: Chlorophyll content of opposite leaves of the third, fifth, and seventh inflorescences after grafting as determined by spectrophotometry. T-SPs, tomato self-grafted plants; Go-tomato, grafted plants with regenerants; GPs, grafted plants. Differences between mean values were analyzed by Fisher’s exact test (*P < 0. 1, ***P < 0.001; n = 9). E, Regenerated buds from Go-tomato plants. F, Go-tomato production in Qinghai, China, in December 2020. G, Yield and fruit quality after grafting. T-SPs, tomato self-grafted plants; Go-tomato, plant with regenerants; GPs, grafted plants. Left to right: Total yield per plant within 6 months after grafting, and anthocyanin content, vitamin C content, and total soluble solids content at 5 months after grafting. Differences between mean values were analyzed by Fisher’s exact test (***P< 0.001; n = 5). Fig. S2. Resequencing of “Go-tomato” (related to Fig. 3). A, Analysis pipeline for whole-genome resequencing. B, Median read density of Go-tomato_PR_1_3 and Go-tomato_LR_1–3 samplesmapped to the goji genome. Window length = 100 kb. Median read density represents count per window length. Fig. S3. Goji DNA fragments transferred to tomato by grafting (related to Fig. 3). A, Mapping results of the [file 43897_2024_124_MOESM1_ESM.zip › 43897_2024_124_Fig8_Print.jpeg]

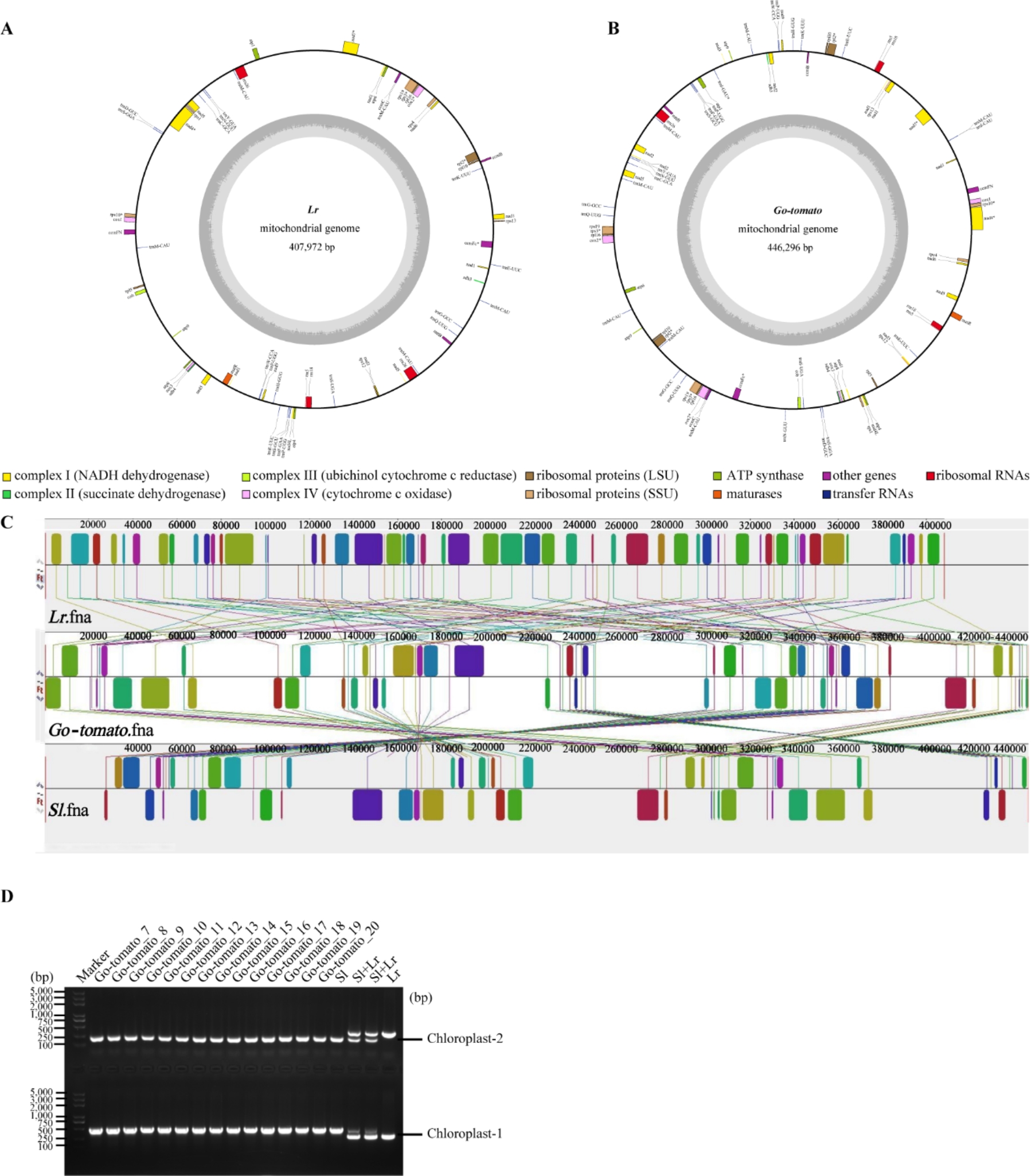

Supplement: Supplementary file 1 — Supplementary Material 1: Fig. S1. Establishment of a grafting system between distant plant species and organogenesis of tomato stem cells (related to Fig. 1). A, Cell wall thickness in goji stems after grafting. Differences between mean values were analyzed by Fisher’s exact test (*P < 0.05;n = 3). B, Survival rate of grafted plants after grafting with goji at the indicated days. Differences between mean values were analyzed by Fisher’s exact test (****P < 0.0001). C, Shoot growth at 80 d after grafting. T-SPs, tomato self-grafted plants; Go-tomato, grafted plants with regenerants at 80 d after grafting; GPs, grafted plants. Scale bar = 10 cm. D, Changes in physiological indices after grafting. Top: Plant height at 10 weeks after grafting. Bottom: Chlorophyll content of opposite leaves of the third, fifth, and seventh inflorescences after grafting as determined by spectrophotometry. T-SPs, tomato self-grafted plants; Go-tomato, grafted plants with regenerants; GPs, grafted plants. Differences between mean values were analyzed by Fisher’s exact test (*P < 0. 1, ***P < 0.001; n = 9). E, Regenerated buds from Go-tomato plants. F, Go-tomato production in Qinghai, China, in December 2020. G, Yield and fruit quality after grafting. T-SPs, tomato self-grafted plants; Go-tomato, plant with regenerants; GPs, grafted plants. Left to right: Total yield per plant within 6 months after grafting, and anthocyanin content, vitamin C content, and total soluble solids content at 5 months after grafting. Differences between mean values were analyzed by Fisher’s exact test (***P< 0.001; n = 5). Fig. S2. Resequencing of “Go-tomato” (related to Fig. 3). A, Analysis pipeline for whole-genome resequencing. B, Median read density of Go-tomato_PR_1_3 and Go-tomato_LR_1–3 samplesmapped to the goji genome. Window length = 100 kb. Median read density represents count per window length. Fig. S3. Goji DNA fragments transferred to tomato by grafting (related to Fig. 3). A, Mapping results of the [file 43897_2024_124_MOESM1_ESM.zip › 43897_2024_124_Fig9_Print.jpeg]
